# Closing the inequality gaps in reproductive, maternal, newborn and child health coverage: slow and fast progressors

**DOI:** 10.1136/bmjgh-2019-002230

**Published:** 2020-01-26

**Authors:** Agbessi Amouzou, Safia S Jiwani, Inácio Crochemore Mohnsam da Silva, Liliana Carvajal-Aguirre, Abdoulaye Maïga, Lara M E Vaz, Countdown 2030 Coverage Technical Working Group, Agbessi Amouzou

**Affiliations:** 1 International Health, Johns Hopkins University Bloomberg School of Public Health, Baltimore, Maryland, USA; 2 International Center for Equity in Health, Universidade Federal de Pelotas, Pelotas, RS, Brazil; 3 Data and Analytics Section, UNICEF, New York, New York, USA; 4 Global Health, Save the Children, Washington DC, District of Columbia, USA

**Keywords:** child health, maternal health

## Abstract

**Introduction:**

Universal Health Coverage (UHC) is a critical goal under the Sustainable Development Goals (SDGs) for health. Achieving this goal for reproductive, maternal, newborn and child health (RMNCH) service coverage will require an understanding of national progress and how socioeconomic and demographic subgroups of women and children are being reached by health interventions.

**Methods:**

We accessed coverage databases produced by the International Centre for Equity in Health, which were based on reanalysis of Demographic and Health Surveys, Multiple Indicator Cluster Surveys and Reproductive and Health Surveys. We limited the data to 58 countries with at least two surveys since 2008. We fitted multilevel linear regressions of coverage of RMNCH, divided into four main components—reproductive health, maternal health, child immunisation and child illness treatment—to estimate the average annual percentage point change (AAPPC) in coverage for the period 2008–2017 across these countries and for subgroups defined by maternal age, education, place of residence and wealth quintiles. We also assessed change in the pace of coverage progress between the periods 2000–2008 and 2008–2017.

**Results:**

Progress in RMNCH coverage has been modest over the past decade, with statistically significant AAPPC observed only for maternal health (1.25, 95% CI 0.90 to 1.61) and reproductive health (0.83, 95% CI 0.47 to 1.19). AAPPC was not statistically significant for child immunisation and illness treatment. Progress, however, varied largely across countries, with fast or slow progressors spread throughout the low-income and middle-income groups. For reproductive and maternal health, low-income and lower middle-income countries appear to have progressed faster than upper middle-income countries. For these two components, faster progress was also observed in older women and in traditionally less well-off groups such as non-educated women, those living in rural areas or belonging to the poorest or middle wealth quintiles than among groups that are well off. The latter groups however continue to maintain substantially higher coverage levels over the former. No acceleration in RMNCH coverage was observed when the periods 2000–2008 and 2008–2017 were compared.

**Conclusion:**

At the dawn of the SDGs, progress in coverage in RMNCH remains insufficient at the national level and across equity dimensions to accelerate towards UHC by 2030. Greater attention must be paid to child immunisation to sustain the past gains and to child illness treatment to substantially raise its coverage across all groups.

**Summary box**

**What is already known?**

- Despite progress in coverage of RMNCH continuum of care since 1990, large disparities remain across women and children groups defined by socioeconomic and demographic status. There is some evidence that progress in overall coverage is occurring faster among the poor and rural populations.
- Understanding how specific socioeconomic and demographic groups are progressing in coverage of RMNCH continuum of care will provide evidence in support of strategies for reaching the Universal Health Coverage (UHC) under the SDGs.

**What are the new findings?**

- Increases in coverage and coverage gaps closed of RMNCH interventions have been modest in the past decade (2008–2017), and mostly noticeable only for reproductive health and maternal health than for child immunisation or illness treatment.
- Considering equity dimensions, older women and those in less well-off groups appear to have progressed substantially faster in coverage of reproductive and maternal health compared with their other counterparts, although the well-off groups still fare much better than the less well-off groups.
- Progress in coverage was not however circumscribed to any particular low-income and middle-income group of countries; fast progress is possible everywhere.

**What do the new findings imply?**

- The current pace of progress in coverage of RMNCH must be accelerated with continued attention to the disadvantaged groups of women and children and specific interventions to close the persistent inequality gaps.
- The slowing trends in child health interventions, especially in child illness treatment, put at serious risk the achievement of UHC, and threaten to offset child survival gains observed in the past decades. Sustained attention must be provided to the child health interventions.

## Introduction

The Sustainable Development Goals (SDGs) for health include a goal of reaching Universal Health Coverage (UHC) by 2030 (goal 3.8).^1^ The goal outlines three key components of UHC, which include financial risk protection; access to quality essential healthcare services; and access to safe, effective, and quality and affordable essential medicine and vaccines for all. The latter two are measured through the coverage of essential health services, encompassing several tracer indicators in reproductive, maternal, newborn and child health (RMNCH), infectious diseases, non-communicable diseases, service capacity and access.^2 3^ Achieving the goal for RMNCH service coverage will require an understanding of national progress in coverage of the RMNCH essential services, and of how well different socioeconomic and demographic subgroups of women and children are being reached by the essential health interventions.^4–6^

Recent RMNCH coverage increases appear to result mainly from faster progress among rural populations or the poor than among the urban or the rich, especially in middle-income countries. A recent study showed that the two poorest quintiles have contributed to accelerate increases in national RMNCH coverage by about 18%.^7^ Similarly, the 2017 global UHC report showed that the percentage of mother-child pairs covered with three or fewer basic health services out of seven declined much faster among the poorest group over the past two decades.^8^ These results are however dependent on baseline coverage levels, given that less educated, rural and poorest populations usually have lower coverage levelsand hence more room for coverage to increase, compared to the educated, urban and richest populations.

Despite substantial progress in the coverage of RMNCH interventions in the past decades, in most low- and middle-income countries (LMICs) large demographic and socioeconomic inequities remain for many interventions. Maternal health indicators such as four or more antenatal care contacts and skilled birth attendant are particularly prone to such inequities, with the rich-poor ratio reaching over fourfold in some countries.^9 10^ Inequities in breast feeding, immunisation and child illness treatment are less pronounced, although the rich are still up to twice more likely to benefit from these interventions than the poor.^11^ Similar inequity patterns are observed by age, with adolescents showing lower coverage of family planning interventions than women aged 20–49 years, and by place of residence. Inequality trend analysis suggests however that the gaps are reducing in most LMICs but at a slow pace.^4 10 12^

Almost 5 years into the SDG era, it is timely to assess which countries and subgroups among women and children are lagging and which are progressing faster. Such analyses will cast light on how countries should target their RMNCH programme to accelerate improvements in coverage and reduce inequities. Evidence indicates that progress in reducing maternal and child mortality across LMICs between 1990 and 2010 was mainly driven by changes in the coverage of health determinants such as health service delivery and immunisation, compounded by demographic and socioeconomic factors.^13^ This paper aims to uncover who have been slow and fast progressors along the RMNCH continuum of care during the past decade (2008–2017), focusing on subgroups defined by age (adolescents, adult women), level of education, place of residence and household wealth quintile, in LMICs with available data. It also compares past and recent progress to assess any acceleration in coverage in the last decade, focusing on trends since the year 2000.

## Methods

### Data

Analysed data for coverage measures come from equity databases produced by the International Centre for Equity in Health (www.equidade.org) at Pelotas University in Brazil. We collaborated with the research team at the centre to obtain additional stratification not included in the standard databases. The databases include RMNCH coverage indicators computed from available nationally representative surveys such as the Demographic and Health Surveys, Multiple Indicator Cluster Surveys and Reproductive Health Surveys carried out during the period 2000–2017. These indicators were computed taking into account the survey sampling weights and stratified cluster sampling design and compared with reported estimates in country reports where possible. Eligible countries were those with at least two such surveys in the past decade (2008–2017), allowing an assessment of coverage change over this period. Among these countries, those with additional data on the period 2000–2007 allowed an assessment of any change in the rate of change in the coverage indicators on the periods 2000–2007 and 2008–2017. A total of 115 countries have data since 2000, including 310 surveys; 105 have surveys since 2008 with a total of 187 surveys. However, only 58 countries had at least two surveys since 2008 with a total of 140 surveys. Of them 52 have a survey on the period 2000–2007. Online supplementary table A1 in the appendix presents the list of countries. The 58 countries include 30% of total LMIC population and account for 66% of maternal deaths and 59% of under-5 years deaths in LMICs.^14–16^ Broken down by country income groups, the study covers 71% of the total population of low-income countries, 37% population of lower middle-income countries and 9% population of upper middle-income countries. Online supplementary map 1 in the appendix shows the distribution of countries included in the analysis.

10.1136/bmjgh-2019-002230.supp1 Supplementary data

### Equity stratifiers

The analysis focused on demographic and socioeconomic groups defined by the age of women (adolescents 15–19, women 20–34 and 35–49 years old), level of maternal education (no schooling, primary, secondary or higher), place of residence (capital city or capital region, other urban, rural) and wealth quintile (poorest quintile, middle three quintiles, richest quintile). All these stratifiers were already available in the original data sets and were collected from interview with each sampled woman during the survey, except for the wealth quintile. The wealth variable was derived from a principal component analysis using information on household assets and characteristics collected during the survey.^17 18^ A score representing the first component was generated, ranked and split into five quintiles representing each 20% of the household population. The bottom quintile represents the poorest 20% of the population while the top quintile represents the richest 20%. For capital cities, some surveys did not distinguish the capital city from the capital region. For these surveys, the urban setting of the capital region was used. In a limited number of countries, the capital city has recently changed to a smaller city. In these cases, the largest city or the previous capital city was used. This was the case for Lagos in Nigeria instead of Abuja and Dar es Salaam in Tanzania instead of Dodoma, Cotonou instead of Porto-Novo in Benin, Abidjan instead of Yamoussoukro in Cote d’Ivoire.

### Coverage measures

We used coverage indicators of four stages of the continuum of care for RMNCH as typically measured in the Composite Coverage Index (CCI). The CCI is a summary measure of coverage indicators along the continuum of care that has been shown to correlate well with measures of health status such child mortality and stunting and used extensively as a valid summary coverage indicator.^19^ We considered separately each of the four components included in the CCI and computed as follows:

1. Reproductive health, measured through the demand for family planning satisfied with modern methods among women of reproductive age, also referred to as family planning coverage.
2. Maternal health, measured as the arithmetic average of coverage of at least four antenatal care visits and skilled birth attendant: MH = (ANC4 +SBA)/2, MH is maternal health coverage, ANC4 is the coverage of four or more antenatal care visits, SBA is coverage of skilled attendant at birth.
3. Child immunisation, measured as a weighted average of DTP3, BCG and measles vaccination: CIm = (2DTP3 +BCG + MSL)/4, where CIm is child immunisation coverage, DTP3 is the coverage of the third dose of diphtheria, tetanus and pertussis vaccine, BCG is the BCG vaccine coverage, MSL is first dose of measles immunisation coverage. DTP3 has a double weight over the other two indicators due to need for three doses.
4. Child illness treatment, measured as the arithmetic average of ORS for diarrhoea and careseeking for acute respiratory infection from a health provider: CIT = (ORS +CAREP)/2 where CIT is child illness treatment, ORS is the coverage of treatment of child diarrhoea with oral rehydration salts solution, CAREP is the careseeking for child’s suspected symptoms of pneumonia.

We analysed separately each of the four components by the equity dimensions to uncover any variation in progress for each specific component. To assess progress in coverage, we used the average annual percentage points change (AAPPC), computed either using multilevel linear regressions to generate cross-country estimates (see below) or using the two extreme survey coverage estimates to generate country-specific estimates.^7^ Given coverage levels are bounded between 0% and 100%, the change in coverage between two time points is often affected by the level at the starting point in such a way that countries with low coverage level have more room to increase coverage than countries with already high coverage level. Furthermore, the AAPPC does not account for the fact that incremental progress in coverage when coverage is already high may require stronger effort than when coverage is low. These limitations can bias comparative inequality trend assessment when absolute increase is used, with groups with higher socioeconomic status tending to show slower progress given their baseline coverage is already high. One way to address this limitation was to use a measure that captures the change in the complement of the coverage indicator to 100%, referred to as the coverage gap. The coverage gap represents the remaining gap to close to reach universal coverage. A relative measure of change in the coverage gap between two times periods shows the proportion of coverage gap that is closed between the two time points. Such measure is a mirror image of the proportion of coverage change but is no longer dependent on the baseline coverage value (see online supplementary box A1 in the appendix). We therefore also computed the proportion of coverage gap closed across countries and for each country and include these results in the online supplementary appendix. Another way of addressing the limitation is controlling for the baseline coverage levels in cases where a regression model is used to assess the annual rate of change.

### Statistical analysis

We described coverage trends in the past 10 years for each of the four continuum of care components defined above. We did this in two ways using indicator data set restricted to countries with at least two coverage estimates on the period 2008–2017. First, at country level by computing the AAPPC between the two extreme surveys on the period 2008–2017. This was done by taking the difference between the two coverage estimates divided by the number of years between the two surveys. Second, at cross-country level, we ran multilevel linear regressions of the coverage level on year to predict the time trend of the coverage across the countries. Two levels were considered, the survey time point as first level and the country as second level. Only a random intercept is used given the limited sample sizes. We predicted trends across all countries included in the analysis as well as within groups of countries based on income grouping, according to the 2019 World Bank classification.^20^ We also ran the multilevel regressions with an interaction between the year and the equity groups to estimate the trends in each specific group separately for each equity stratifier. Given coverage measures are between 0% and 100%, the application of linear models can be limited due to the possibility of predictions beyond these bounds. An alternative is to run the linear model on a logit transformed of the coverage variable.^21^ However, such transformation yields regression coefficients that are not easily interpretable. This approach is most useful if interest is in predicted estimates which can be back transformed to original values between 0% and 100%. We have preferred the linear model over a logit transformed model because it generates coefficients that are easily interpretable as annual coverage change and we were not interested in predictions beyond the period for which data were available. We also confirmed that the two types of models are identical by running and comparing the predicted coverage estimates using the linear model and the logit transformed (see online supplementary appendix figure A2).

To control the effects of starting coverage levels in the trend analysis, we included in the linear model the coverage level of the initial survey. We also computed the annual per cent of coverage gap closed on the same period (2008–2017). Cross-country averages and 95% CIs from the regressions as well as country-specific estimates are produced to quantify overall progress in each equity dimension and for each continuum of care component.

For countries with at least two data points on the period 2008–2017, and at least another point on the prior period 2000–2007, we assessed whether there had been any change in the pace of progress in coverage change by fitting a multilevel regression model on the period 2000–2017 and including a spline knot in 2008. The spline regression approach with a knot in 2008 allowed distinguishing the coverage trends between the periods 2000–2007 and 2008–2017. The data set included countries with at least three coverage estimates on the period 2000–2017, at least two of which must be on the period 2008–2017. AAPPC on the periods 2000–2007 and 2008–2017 across all countries and for each equity dimension were generated and compared to assess any acceleration, stagnation or deceleration in the pace of coverage change. All statistical analyses were carried out using Stata V.14.1.

## Results

### Progress in RMNCH coverage in the past decade (2008–2017)

**Figure 1:**
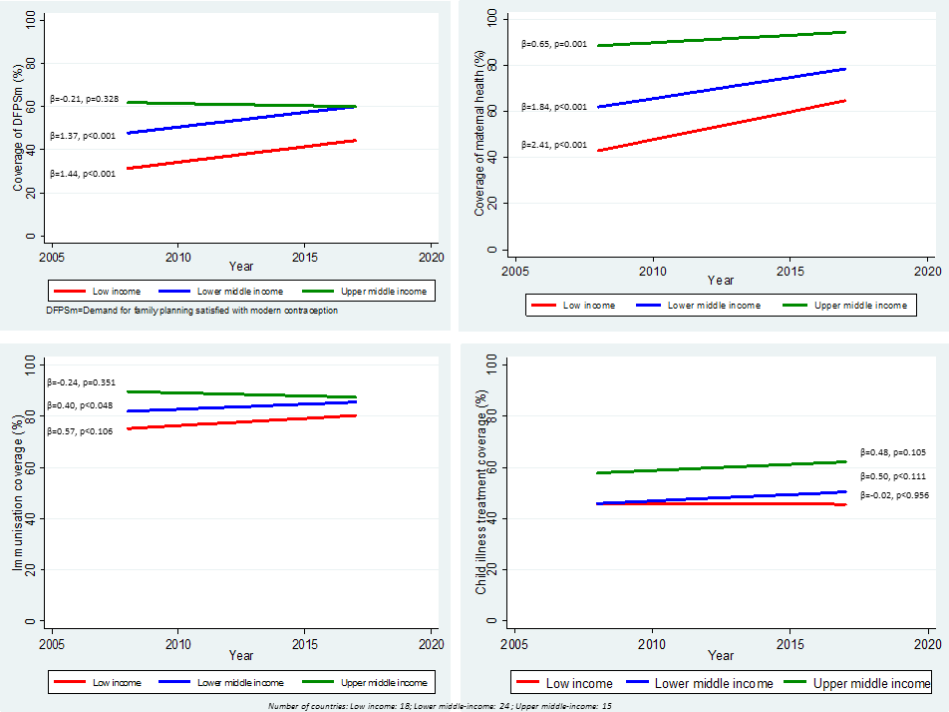
Trends in coverage of four components of the RMNCH along the continuum of care on the period 2008-2017 by countries categorized by income group. (β represents the slope of the line, p is p-value associated with the slope).

Overall, increases in coverage of RMNCH interventions have been modest in the past decade (2008–2017) for the four components of the continuum of care analysed. Figure 1 presents the crude AAPPC in the coverage of each of these components, using multilevel linear regression and disaggregated by World Bank country income group classification (June 2019 version).^20^ Corresponding scatter plots with predicted trend lines are shown in the online supplementary appendix figure A3. Only the AAPPC in maternal health coverage shows a statistically significant trend for the three income groups, with the largest AAPPC of about two percentage points per year observed in low income countries (AAPPC=2.41, p<0.001) and lower middle-income countries (AAPPC=1.84, p<0.001). The upper middle-income country group, with already high coverage, increased significantly by 0.65 (p<0.001) percentage points per year. Statistically significant average increases of more than one percentage point per year were also noted for reproductive health among low and lower middle-income countries; however, no statistically significant coverage change is observed for the upper middle-income group. Progress in coverage has been marginal for child immunisation coverage across all groups, where coverage levels were generally already high, and child illness treatment coverage, where the annual change was small and not statistically significant in any of the three groups.

**Figure 2:**
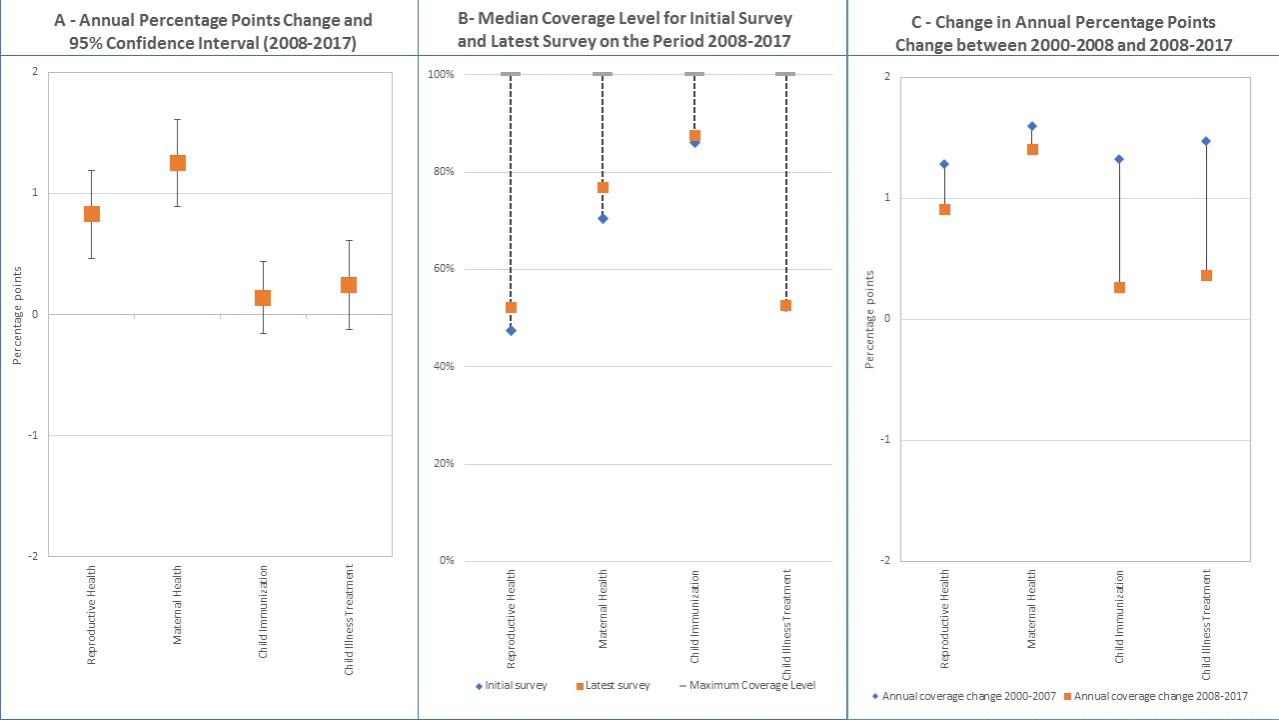
Levels and changes in national coverage for RMNCH continuum of care.

Figure 2A presents the AAPPC and its 95% CIs, controlling for the starting coverage value in each country, for each of the four continuum of care components. Online supplementary appendix table A2 in the appendix presents actual coefficients, their statistical significance level, and CIs based on multilevel linear regressions of coverage. Consistent with the previous results, the fastest and statistically significant progress is observed for maternal health with an AAPPC of 1.25 (95% CI 0.90 to 1.61), ahead of progress for reproductive health for which the AAPPC is only 0.83 (95% CI 0.47 to 1.19). The AAPPC was not statistically significant for child immunisation and child illness treatment. The figure 2B shows actual median coverage levels from the two extreme surveys on the period and for each continuum of care component. The dotted lines visually display the existing gap that needs to be closed from the initial survey and how median coverage progressed in the latest survey. The chart confirms the observed progress in maternal and reproductive health and small change or no change in child immunisation and child illness treatment. However, coverage levels vary across these four components with highest coverage observed for child immunisation (86% for the initial surveys and 88% for the latest), followed by maternal health (70% and 77%). Coverage for reproductive health and child illness treatment is lowest at around 50%. Figure 2C compares annual percent coverage change between the periods 2000–2007 and 2008–2017 derived from the multilevel linear regression with spline knot at the year 2008. The corresponding table with coefficients and CIs is presented in the online supplementary appendix table A3. Corresponding scatterplots with predicted trends are shown in online supplementary appendix figure A4. It shows that overall progress in coverage did not accelerate in the recent decade for all four components of the continuum of care, and in fact, there was substantially larger reduction in the pace for child immunisation and child illness treatment than for reproductive and maternal health. AAPPC reduced from 1.28 to 0.92 for reproductive health and from 1.6 (95% CI 1.09 to 2.12) to 1.41 (95% CI 1.01 to 1.80) for maternal health. For child immunisation and child illness treatment, the trends reduced from 1.31 (95% CI 0.91 to 1.74) to 0.27 (95% CI−0.04 to 0.58) and from 1.47 (95% CI 1.01 to 1.93) to 0.37 (95%CI 0.03 to 0.71), respectively. When countries are divided by income groups, some differential results emerged for trends in reproductive and maternal health (online supplementary table A3 in the appendix). Countries in the low-income group appear to have accelerated coverage change for maternal health while a lack of acceleration was observed for the other groups. Similarly, countries in the lower middle-income group have accelerated changes in coverage of reproductive health compared with other groups. When the average annual per cent of coverage gap closed is used (online supplementary appendix figure A9), the results suggest that a slightly higher coverage gap was closed on the most recent decade compared with the previous for reproductive and maternal health, while the proportion of gap closed declined for child immunisation and illness treatment.

**Figure 3:**
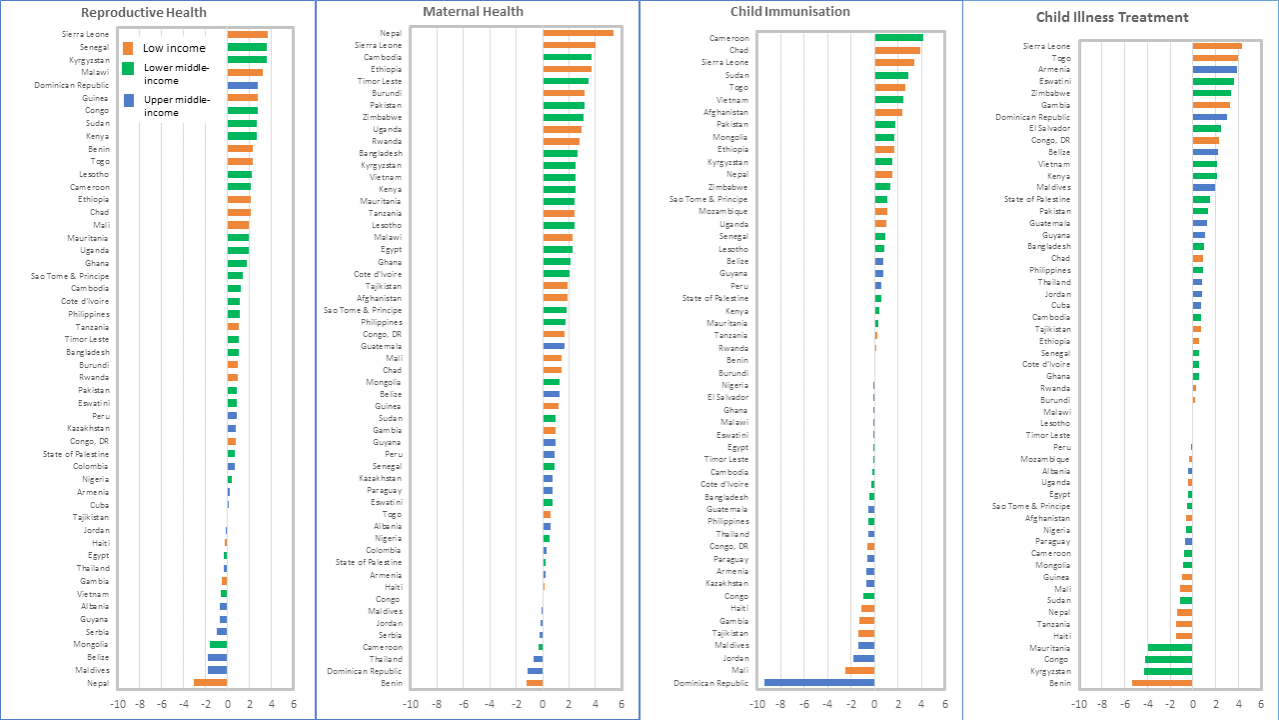
Average annual percentage points changes (AAPPC) in coverage on the period 2008-2017 by country for each of the four components of RMNCH continuum of care.

Figure 3 shows the distribution of the AAPPC in coverage by country for each component of the continuum of care for the period 2008–2017, with country highlighted by their income group classification. The corresponding average coverage gap closed by country is included in the online supplementary appendix figure A10. This distribution allows an analysis of countries that are fast progressors and those that are trailing at the bottom. There is a great deal of diversity between the fast and slow progressors. AAPPC varies from −3.1 to 4.6 for reproductive health, from −1.2 to 5.3 for maternal health, from −9.3 to 4.1 for child immunisation and from −5.4 to 4.3 for child illness treatment. For reproductive and maternal health, a handful of countries experienced coverage decline or stagnation in the past decade while about half of the countries experienced decline in child immunisation and child illness treatment coverage. Furthermore, countries at the top of the rank vary widely across the four components. Except for Sierra Leone which is in the top 10 countries with the fastest progress for at least three of the four components, all other top 10 countries rank high only for one or two components. Moreover, there are countries from all continents represented among the fast progressors, from Africa to Latin America and the Caribbean. This underscores the evidence that progress, and fast progress, was not circumscribed to any particular continent but was spread all across. Similarly, the slowest progressor countries also vary across the LMIC continents. Except for Thailand, which experienced coverage decline for three of the four components, most other countries at the bottom of the distribution experienced coverage decline for only one or two components. Particularly, the Dominican Republic was in the top 10 countries with the fastest progress for reproductive and child illness treatment but dropped in the bottom 10 with coverage decline for maternal health and child immunisation coverage.

**Figure 4:**
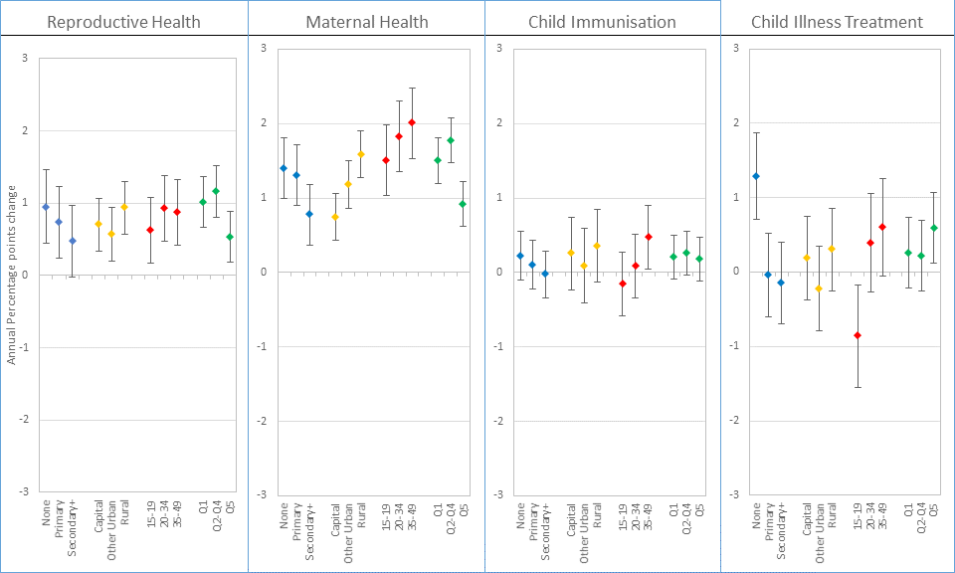
Average annual percentage points changes (AAPPC) in coverage for the components of the continuum of care on the period 2008-2017 for groups defined according to education, place of residence, maternal age, and wealth quintiles.

**Figure 5:**
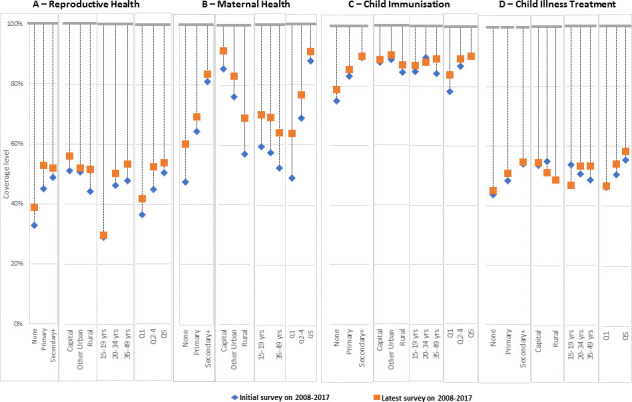
Absolute coverage at initial and latest survey for the components of the continuum of care on the period 2008-2017 for groups defined according to education, place of residence, maternal age, and wealth quintiles.

When the analysis is disaggregated by equity dimensions, progress varies based on the type of interventions and the equity group. Figure 4 shows the AAPPC on the period 2008–2017 derived from the multilevel linear regression, controlling for the starting coverage values. The actual regression results are included in the online supplementary appendix A2. Median and IQR of the annual per cent of coverage gap closed over the period 2008–2017 for each RMNCH component and for equity groups are shown in the online supplementary appendix figure A11. To appreciate the coverage levels and the size of the coverage gap by equity groups, figure 5 shows the gap between the overall median coverage between the initial survey and the latest survey over the period 2008–2017. Online supplementary appendix pages 12–19 shows country-specific AAPPC. We describe the results specifically for each component of the RMNCH continuum of care.

### Reproductive health

While overall progress in reproductive health has been modest over the period 2008–2017, some groups have progressed more slowly than others, judging by the AAPPC on figure 4. Women with no education, in rural areas, aged 20 years or more, and in the poorest or middle two to four quintile groups appeared to have experienced faster progress in coverage. Conversely, women with secondary or plus education, adolescents and those in the richest quintiles appeared the slowest. The overlapping 95% CIs of the AAPPCs suggest that the differential trends were not statistically significant across the equity dimensions, although taken individually, the coverage trends were all statistically significant, except for women with secondary or more education for whom the trend was only marginally significant. The coverage gap closed figure (online supplementary appendix figure A11) shows attenuated differentials across the group. In terms of the absolute coverage gap, adolescents showed the highest coverage gap that was stagnant over this period (figure 5).

### Maternal health

Maternal health is the component that experienced the fastest progress in coverage change. While coverage trends were statistically significant in all equity groups, the pace varied substantially (figure 4). Fastest progress was observed among women aged 20–49 years. The slow movers included women in the secondary or more education group, in the capital city and those in the richest quintile. Here again, women in the poorest or middle quintiles appeared to have progressed substantially much faster than those in the richest quintile. Similarly, women in rural areas experienced faster progress than those in the capital city.

### Child immunisation

Average trends in immunisation coverage have been the slowest of the four components of the continuum of care analysed for the period 2008–2017. Child immunisation coverage is generally high, thus has narrow room for improvements. The coverage trends were statistically significant only among women aged 35–49 years, who were the fastest progressors with AAPPC of 0.47 (95% CI 0.05 to 0.90) (figure 4).

### Child illness treatment

Similar to child immunisation, child illness treatment coverage did not show substantial progress in the past decade. Statistically significant positive trends were observed among women with no education and those in the richest quintile, with respectively 1.29 (95% CI 0.71 to 1.87) and 0.60 (95% CI 0.12 to 1.07) annual percentage points increase in coverage. The slowest progressors were the adolescents, which experienced a reversal of coverage with a statistically significant decline annual percentage point of −0.86 (95% CI −1.55 to 0.18).

### Comparing past and recent trends

**Figure 6:**
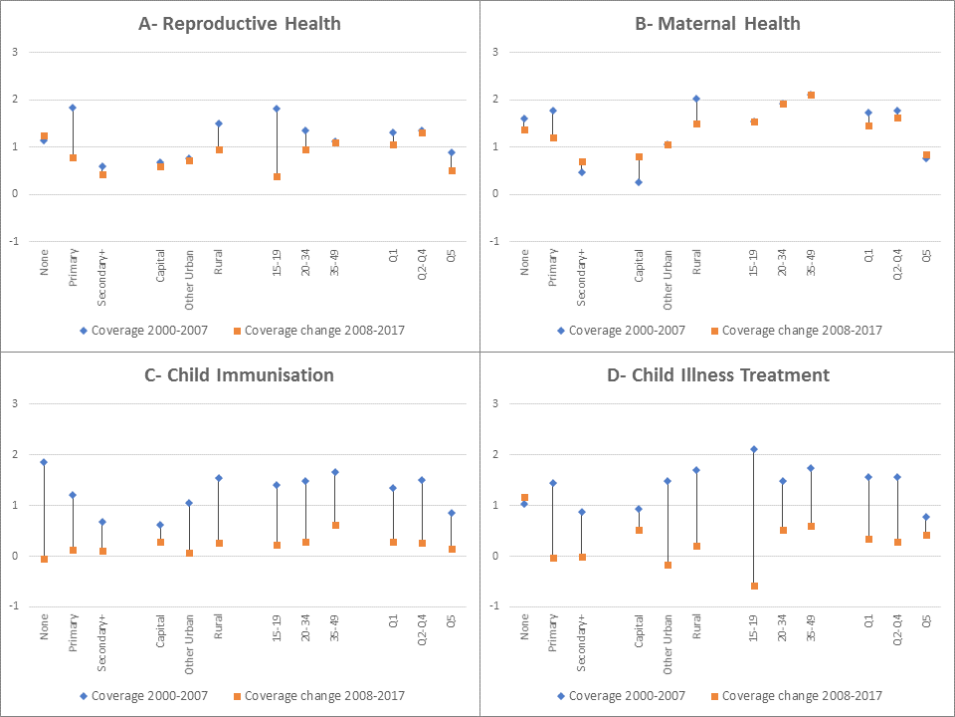
Change in the average annual percentage points change of coverage between periods of 2000-2007 and 2008-2017 for each component of the continuum of care.

Figure 6 compares the AAPPC between the periods 2000–2008 and 2008–2017 to assess any acceleration or deceleration in coverage change. The regression coefficients and CIs are included in the online supplementary appendix table A3. For reproductive and maternal health, there appeared no acceleration of the coverage trends in the past decade compared with the previous. Instead a deceleration in reproductive health coverage is observed among women with primary education (AAPPC went from 1.83 to 0.77) and among adolescents (AAPPC went from 1.81 to 0.39). Reduction in the coverage trends was much more pronounced for child immunisation and child illness treatment, where coverage increase in almost all equity groups appeared to have slowed down substantially. The largest deceleration in child immunisation was observed among women with no education (AAPPC went from 1.86 during 2000–2008 to −0.04 during 2008–2017). For child illness treatment, children of adolescent mothers appeared affected more severely by the deceleration, going from 2.1 percentage points increase during the period 2000–2008 to an annual decline of −0.58 percentage points during 2008–2017.

## Discussion

While LMICs strategise for UHC and the SDGs, assessment of progress in RMNCH intervention coverage in the past two decades yields sobering results. Substantial inequalities in RMNCH coverage across socioeconomic and demographic groups of women and children remain. Although the inequality gaps are closing, current national coverage changes are too slow to generate the needed acceleration toward UHC goals by 2030. We analysed four components of the continuum of care for RMNCH, including reproductive health, maternal health, child immunisation and child illness treatment. Each component was measured by a summary measure of a limited number of relevant indicators, except for reproductive health which was indicated by the proportion of demand for family planning that was satisfied with modern contraception. Across 58 countries with multiple surveys available since 2008, progress in coverage of RMNCH interventions has been modest over the period 2008–2017, and mostly noticeable only for reproductive health and maternal health than for child immunisation or illness treatment. We found that coverage of reproductive and maternal health increased significantly by 0.83 (95% CI 0.47 to 1.19) and 1.25 (95% CI 0.90 to 1.61) percentage points, respectively, over the past decade while such trend was not statistically significant for child immunisation (at 0.14 percentage points (95% CI −0.16 to 0.44)) and child illness treatment (at 0.25 percentage points (95% CI −0.12 to 0.61)). Although these trend estimates controlled for the starting coverage values, the slower and non-significant pace of child immunisation may be in part due to the already high coverage for this component. Median child immunisation coverage for the latest survey was 88%, compared with 52% for reproductive health and 77% for maternal health. However, this was not the case for child illness treatment, for which median coverage was 53% and no statistically significant increase was observed in the past decade. Despite major programmes being deployed at facility and community levels, effort in reaching children with common, yet fatal, illnesses such as diarrhoea and pneumonia must identify the best strategies to increase utilisation of services and access to life-saving interventions.^22–24^

Progress in RMNCH coverage in the past decade also differed by country income group level and the intervention component. Faster annual coverage increases were observed in low and lower middle-income countries compared with upper middle-income countries for reproductive and maternal health. Statistically significant, but modest annual coverage increases (0.38 percentage points (95% CI 0.02 to 0.73)) were also observed among lower middle-income countries for child immunisation while the trends were not statistically significant in other groups. The fastest trends in low and lower-middle income countries are confirmed in other earlier studies that found that coverage of RMNCH interventions was progressing much faster in these groups than in the upper middle-income countries^7 12^

While there is large variability across countries in coverage progress, there was not any group of countries in a particular region of the world that consistently showed fast progress across the RMNCH continuum of care. The exceptions were Sierra Leone, which was among the top 10 countries with positive increases in coverage of all four RMNCH components included in the analysis, and Kyrgyzstan, which showed positive performance in three of the four. Fast progressors were spread throughout all continents, in Africa, Asia, Eastern Europe, and Latin America and the Caribbean. Similarly, there was no regional group of slowest progressors. This variability in countries highlights the fact that both fast progress and impediments to coverage are spread across all continents, and impediments can be overcome.

The assessment of fast and slow progressors in terms of equity dimensions revealed a consistent pattern for maternal and reproductive health. While increasing trends in coverage of reproductive and maternal health were statistically significant on the past decades, some groups have moved faster than others. In general, it appeared that socioeconomically less well-off groups and older women appeared to have moved faster. For reproductive health, women with no education, in rural areas, aged 20 years or more, and in the poorest or middle two to four quintile groups experienced faster progress in coverage, while women with secondary or plus education, adolescents and those in the richest quintiles appeared the slowest. For maternal health, fastest progress was observed among women aged 20–49 years, while women in the secondary or more education group, in the capital city and those in the richest quintile were the slow movers. For child immunisation, there was large variability across the groups with generally non-statistically significant progress, except among women aged 35–49 years. For child illness treatment, adolescents were the slowest while women with no education showed the fastest progress. Overall, the analysis showed that traditionally well-off groups such as the richest quintiles, educated women or those in urban areas have not progressed consistently faster in RMNCH and in fact, are being caught up and in many cases outperformed by the poorest and rural women. These well-off groups remain nevertheless better-off in terms of coverage of RMNCH. This result is consistent with earlier studies that have shown that increases in absolute coverage are largest among the poor or rural population than among the richest and urban populations, a reflection of lower coverage levels among the former than among the latter.

During this early period of the SDGs, expected accelerations in the RMNCH coverage, compared with earlier decade, are not met across all components of the continuum of care. Assessment of the annual coverage change across the board showed no acceleration in coverage between 2000–2007 and 2008–2017. Trends appeared to have slightly slowed down for reproductive and maternal health, although when the coverage gap closed is assessed, a higher proportion of coverage gap appeared to have been closed for these two components. The most striking was the pervasive deceleration observed for child immunisation and child illness treatment. These slowing trends put at serious risk the achievement of UHC and threaten to offset child survival gains observed in the past decades.

The analyses presented have some limitations. First, the analysis was limited only to countries with multiple available surveys of the period 2008–2017, which were in total 58 countries. These countries cover only 30% of the LMICs but account for two-thirds of maternal deaths and close to 60% of child deaths in LMICs. They should not be considered as representative of all the countries. This is particularly the case when the countries were separated by the income groups and only 9% of the total population in upper middle income was covered by countries included in the analysis. Second, our measures of each of the four components of RMNCH do not encompass all indicators in each domain, nor do they include all stages of the continuum of care. For example, indicators considered for maternal health—at least four antenatal care visits and skilled birth attendant—only measure contact with the health system and do not capture all content interventions required in antenatal and delivery care. These indicators also do not capture the quality of care provided. The fastest progress observed in these indicators may reflect this feature and conceal any lack of progress on key content interventions. Furthermore, it does not capture newborn health although it is generally assumed that interventions captured under maternal health affect neonatal health outcomes. Similarly, child illness treatment was measured only through a combination of diarrhoea treatment with ORS and child careseeking for symptoms of acute respiratory infections. Nevertheless, our choice of indicators was consistent with the set of indicators used in the CCI and the 2017 UHC report.^8^ Third, sample sizes for the analysis were limited given the limited number of countries and surveys. Furthermore, we used computed coverage indicators in the multilevel analysis without accounting for the within country or group standard errors of these coverage indicators. Although the standard errors of the individual coverage indicator were available, the computation of the standard errors of three of the four composite indicators analysed (maternal health, child immunisation and child illness treatment) would have required accessing the individual level records of each single survey and using non-parametrical methods such as jackknife or bootstrapping due to collinearity between indicators included in each composite indicator. Finally, the analysis was not weighted by country populations as we were not seeking to generate population representative global or regional estimates of coverage changes but to focus on trends at country level themselves.

Our findings nevertheless provide evidence that current coverage trends do not yet demonstrate acceleration towards achieving UHC and call for stronger and more effective strategies to equalise coverage levels across groups and rapidly increase coverage to achieve UHC. These strategies should address how to sustain the effectiveness of vertical programmes such as child immunisation and how to adopt a continuum of care approach that integrates more comprehensively and effectively services, while accounting for the needs and demand of populations. While the four components analysed are strongly related within the continuum of care framework, related programmes are often implemented and assessed separately. The low and stagnant coverage of child illness treatment contrasts surprisingly with the large efforts to reach women and children through community delivery programmes such as the integrated community case management of childhood illnesses, suggesting that current strategies needs careful review and fine-tuning. Finally, progress assessment in coverage and UHC is predicated on the availability of regular national data that can be disaggregated according to socioeconomic and demographic status as well as subnational groups.
