# Supplementary material for: Closing the inequality gaps in reproductive, maternal, newborn and child health coverage: slow and fast progressors
Source: BMJ Glob Health. 2020 Jan 26;5(1):e002230. doi: 10.1136/bmjgh-2019-002230 (PMC7042586; doi:10.1136/bmjgh-2019-002230)

 BMJ Publishing Group Limited (BMJ) disclaims all liability and responsibility arising from any reliance placed on this supplemental material which has been supplied by the author(s)

Table A1: List of countries with at least two surveys on the period 2008-2017 included in the analysis

country	Income Level (World Bank 2019)	year1	source1	year2	source2	year3	source3	year4	source4	year5	source5	year6	source6	year7	source7	year8	source8	year9	source9
Afghanistan[+]	Low	2010	MICS	2015	DHS														
Albania	Upper middle	2002	RHS	2005	MICS	2008	DHS	2017	DHS										
Armenia	Upper middle	2000	DHS	2005	DHS	2010	DHS	2015	DHS										
Bangladesh	Lower middle	1993	DHS	1996	DHS	1999	DHS	2004	DHS	2006	MICS	2007	DHS	2011	DHS	2012	MICS	2014	DHS
Belize	Upper middle	1991	RHS	2006	MICS	2011	MICS	2015	MICS										
Benin	Low	1996	DHS	2001	DHS	2006	DHS	2011	DHS	2014	MICS								
Burundi	Low	2005	MICS	2010	DHS	2016	DHS												
Cambodia	Lower middle	2000	DHS	2005	DHS	2010	DHS	2014	DHS										
Cameroon	Lower middle	1998	DHS	2004	DHS	2006	MICS	2011	DHS	2014	MICS								
Chad	Low	1996	DHS	2004	DHS	2010	MICS	2014	DHS										
Colombia	Upper middle	1995	DHS	2000	DHS	2005	DHS	2010	DHS	2015	DHS								
Congo Brazzaville	Lower middle	2005	DHS	2011	DHS	2014	MICS												
Congo Democratic Republic	Low	2007	DHS	2010	MICS	2013	DHS												
Cote d'Ivoire	Lower middle	1994	DHS	1998	DHS	2006	MICS	2011	DHS	2016	MICS								
Cuba	Upper middle	2006	MICS	2010	MICS	2014	MICS												
Dominican Republic	Upper middle	1996	DHS	1999	DHS	2002	DHS	2007	DHS	2013	DHS	2014	MICS						
Egypt	Lower middle	1995	DHS	2000	DHS	2005	DHS	2008	DHS	2014	DHS								
El Salvador	Lower middle	1993	RHS	1998	RHS	2002	RHS	2008	RHS	2014	MICS								
Eswatini	Lower middle	2006	DHS	2010	MICS	2014	MICS												
Ethiopia	Low	2000	DHS	2005	DHS	2011	DHS	2016	DHS										
Gambia	Low	2005	MICS	2010	MICS	2013	DHS												
Ghana	Lower middle	1993	DHS	1998	DHS	2003	DHS	2006	MICS	2008	DHS	2011	MICS	2014	DHS				
Guatemala	Upper middle	1995	DHS	1998	DHS	2002	RHS	2008	RHS	2014	DHS								
Guinea	Low	1999	DHS	2005	DHS	2012	DHS	2016	MICS										
Guyana	Upper middle	2006	MICS	2009	DHS	2014	MICS												
Haiti	Low	1994	DHS	2000	DHS	2005	DHS	2012	DHS	2016	DHS								
Jordan	Upper middle	1997	DHS	2002	DHS	2007	DHS	2012	DHS	2017	DHS								
Kazakhstan	Upper middle	1995	DHS	1999	DHS	2006	MICS	2010	MICS	2015	MICS								
Kenya	Lower middle	1993	DHS	1998	DHS	2003	DHS	2008	DHS	2014	DHS								
Kyrgyzstan	Lower middle	1997	DHS	2005	MICS	2012	DHS	2014	MICS										
Lesotho	Lower middle	2004	DHS	2009	DHS	2014	DHS												
Malawi	Low	2000	DHS	2004	DHS	2006	MICS	2010	DHS	2013	MICS	2015	DHS						
Maldives[+]	Upper middle	2009	DHS	2016	DHS														
Mali	Low	1995	DHS	2001	DHS	2006	DHS	2009	MICS	2012	DHS	2015	MICS						
Mauritania	Lower middle	2007	MICS	2011	MICS	2015	MICS												
Mongolia	Lower middle	2005	MICS	2010	MICS	2013	MICS												

Mozambique	Low	1997	DHS	2003	DHS	2008	MICS	2011	DHS	2015	DHS								
Nepal	Low	1996	DHS	2001	DHS	2006	DHS	2010	MICS	2011	DHS	2014	MICS	2016	DHS				
Nigeria	Lower middle	1999	DHS	2003	DHS	2007	MICS	2008	DHS	2011	MICS	2013	DHS	2016	MICS				
Pakistan	Lower middle	2006	DHS	2012	DHS	2017	DHS												
Paraguay	Upper middle	1995	RHS	1998	RHS	2004	RHS	2008	RHS	2016	MICS								
Peru	Upper middle	1996	DHS	2000	DHS	2004	DHS	2005	DHS	2006	DHS	2007	DHS	2008-2016	DHS*				
Philippines	Lower middle	1993	DHS	1998	DHS	2003	DHS	2008	DHS	2013	DHS	2017	DHS						
Rwanda	Low	2000	DHS	2005	DHS	2010	DHS	2014	DHS										
Sao Tome and Principe+	Lower middle	2008	DHS	2014	MICS														
Senegal	Lower middle	1997	DHS	2005	DHS	2010	DHS	2012	DHS	2014	DHS	2015	DHS	2016-2016	DHS*				
Serbia	Upper middle	2005	MICS	2010	MICS	2014	MICS												
Sierra Leone	Low	2005	MICS	2008	DHS	2010	MICS	2013	DHS										
State of Palestine+	Lower middle	2010	MICS	2014	MICS														
Sudan	Lower middle	2010	MICS	2014	MICS														
Tajikistan	Low	2005	MICS	2012	DHS	2017	DHS												
Tanzania	Low	1996	DHS	1999	DHS	2004	DHS	2010	DHS	2015	DHS								
Thailand	Upper middle	2005	MICS	2012	MICS	2015	MICS												
Timor Leste+	Lower middle	2009	DHS	2016	DHS														
Togo	Low	1998	DHS	2006	MICS	2010	MICS	2013	DHS										
Uganda	Low	1995	DHS	2000	DHS	2006	DHS	2011	DHS	2016	DHS								
Vietnam	Lower middle	1997	DHS	2002	DHS	2006	MICS	2010	MICS	2013	MICS								
Zimbabwe	Lower middle	1994	DHS	1999	DHS	2005	DHS	2009	MICS	2010	DHS	2014	MICS	2015	DHS				

* Continuous annual surveys

+ countries with data since 2008

Box A1: Measuring progress in coverage: the coverage gap closed
A challenge that studies assessing comparative progress in coverage measure often face, and don't usually address adequately, is the dependency of the pace of change on the starting or baseline level of the coverage measures. Typically, it is much more difficult to improve coverage rates that are already high than to do so for coverage rates that are low, the latter having more room to increase in absolute terms than the former. Thus, using absolute or relative change in coverage levels is subject to these limitations and it is essential to control for baseline values. One way to do this is to use a measure based on coverage gap, that is, the difference between the coverage level and the maximum coverage level of 100%. The *relative percent of coverage gap closed* between two time points expresses the progress in filling up the gap between the two time points, expressed in relative terms based on the gap at the initial time point. It is computed as: $$\%CGC = 100 * (1 - \frac{100 - C_2}{100 - C_1})$$ Where C1 is coverage level at time 1 and C2 is coverage level at time 2, both expressed in percentage. The %CGC therefore is a relative measure that captures progress made in a closing the remaining coverage gap to reach 100%. For comparison across countries, the %CGC can be annualized by dividing it by the number of years between the two time points. <br>Analyzing the coverage gap that has been closed allows controlling for the baseline coverage value. To illustrate this point, consider two population groups A and B, and assume A progressed from a coverage level of 20% to 60% between times t1 and t2, and B went from 80% to 90% over the same period. The absolute coverage change in A and B is respectively 40 (=60%-20%) and 10 (=90%-80%) percentage points, suggesting higher coverage change in A than B. Similarly, the relative coverage change in A and B is 200% (=(60-20)/20) and 12.5% (=(90-80)/80). However, these results do not account for the fact that A has very low coverage level compared to B. Considering the coverage gap approach, the coverage gap closed between times t1 and t2 by group A is (80-40)/80 =50%, and by group B is (20-10)/20=50%. This shows that the two groups have closed coverage gaps by the same relative size. The analysis, however, assumes that coverage is not decreasing substantially in both groups, which is usually the case for most groups. However, by equating a large jump in coverage from a low level to a high level (say from 20% to 60%) to a modest jump from an already high level (say from 80% to 90%) this measure may qualitatively imply similar health improvements. We recommend therefore presenting this measure along with the actual coverage change indicator. See further details in the discussion section.

Figure A1: Countries included in the analysis

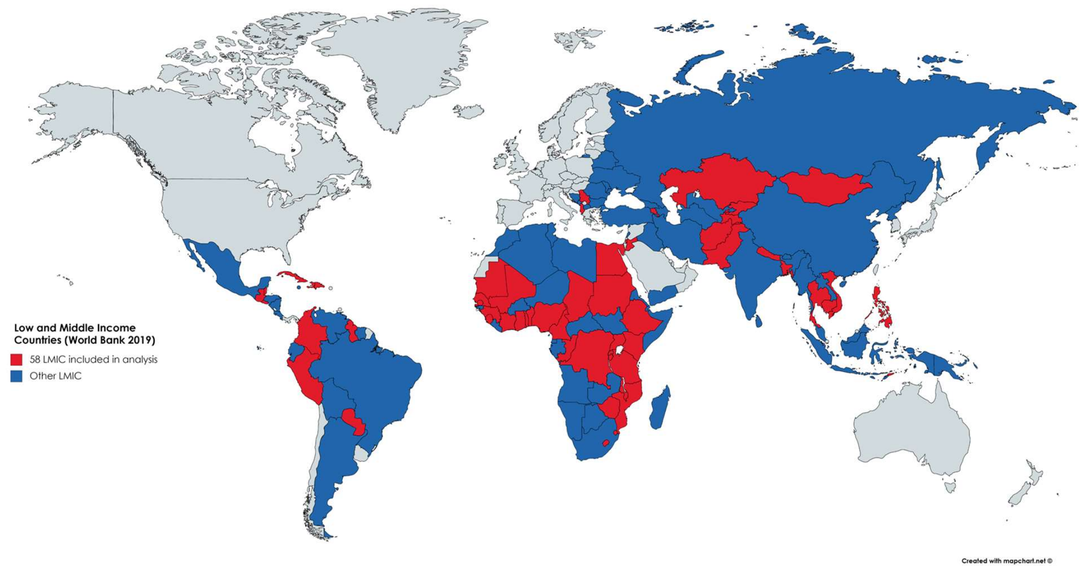

Amouzou A, *et al. BMJ Global Health* 2020; 5:e002230. doi: 10.1136/bmjgh-2019-002230

Table A2: Annual percentage points change in coverage (coeff) of each of the four components of the continuum of care for RMNCH on the period 2008-2017 (models adjusted for starting coverage value)

Category	Reproductive Health				Maternal health				Child immunization				Child illness treatment			
	n	Coeff	signif	95%CI	n	Coeff	signif	95%CI	n	Coeff	signif	95%CI	n	Coeff	signif	95%CI
All countries (58)	130	0.83	**<0.001***	(0.47 , 1.19)	135	1.25	**<0.001***	(0.90 , 1.61)	129	0.14	0.359	(-0.16 , 0.44)	134	0.25	0.19	(-0.12 , 0.61)
Low income countries (19)	41	1.21	**0.003***	(0.41 , 2.01)	43	2.06	**<0.001***	(1.31 , 2.81)	42	0.29	0.424	(-0.42 , 0.99)	44	-0.34	0.348	(-1.07 , 0.38)
Lower middle income countries (24)	56	1.36	**<0.001***	(0.90 , 1.82)	57	1.57	**<0.001***	(1.07 , 2.07)	58	0.38	**0.036***	(0.02 , 0.73)	59	0.48	0.108	(-0.11 , 1.07)
Upper middle income countries (15)	33	-0.23	0.277	(-0.64 , 0.18)	35	0.58	**<0.001***	(0.34 , 0.82)	29	-0.32	0.192	(-0.79 , 0.16)	31	0.47	0.108	(-0.10 , 1.05)
Education	384				395				375				388			
None		0.95	**<0.001***	(0.45 , 1.46)		1.40	**<0.001***	(0.99 , 1.81)		0.22	0.173	(-0.10 , 0.55)		1.29	**<0.001***	(0.71 , 1.87)
Primary		0.74	**0.004***	(0.24 , 1.23)		1.31	**<0.001***	(0.90 , 1.72)		0.10	0.527	(-0.22 , 0.43)		-0.04	0.89	(-0.60 , 0.52)
Secondary or more		0.48	0.058	(-0.02 , 0.97)		0.78	**<0.001***	(0.37 , 1.18)		-0.03	0.864	(-0.35 , 0.29)		-0.15	0.595	(-0.70 , 0.40)
Place of residence	388				385				361				374			
Capital city/region		0.70	**<0.001**	(0.34 , 1.07)		0.74	**<0.001***	(0.43 , 1.06)		0.25	0.312	(-0.24 , 0.74)		0.18	0.521	(-0.38 , 0.74)
Other urban		0.57	**0.002***	(0.20 , 0.94)		1.19	**<0.001***	(0.87 , 1.50)		0.09	0.725	(-0.41 , 0.59)		-0.22	0.438	(-0.79 , 0.34)
Rural		0.94	**<0.001**	(0.57 , 1.30)		1.59	**<0.001***	(1.27 , 1.90)		0.36	0.151	(-0.13 , 0.85)		0.30	0.283	(-0.25 , 0.86)
Maternal age	378				147				198				200			
15-19		0.63	0.007	(0.18 , 1.08)		1.50	**<0.001***	(1.03 , 1.98)		-0.15	0.479	(-0.58 , 0.27)		-0.86	**0.014***	(-1.55 , -0.18)
20-34		0.93	**<0.001***	(0.48 , 1.39)		1.82	**<0.001***	(1.35 , 2.30)		0.09	0.688	(-0.34 , 0.51)		0.39	0.242	(-0.26 , 1.05)
35-49		0.87	**<0.001***	(0.42 , 1.32)		2.00	**<0.001***	(1.53 , 2.48)		0.47	**0.029***	(0.05 , 0.90)		0.60	0.073	(-0.06 , 1.26)
Wealth quintile	384				405				387				396			
Poorest (q1)		1.01	**<0.001***	(0.66 , 1.37)		1.50	**<0.001***	(1.20 , 1.81)		0.20	0.175	(-0.09 , 0.50)		0.26	0.278	(-0.21 , 0.74)
Middle (q2-q4)		1.16	**<0.001***	(0.81 , 1.52)		1.78	**<0.001***	(1.47 , 2.08)		0.26	0.082	(-0.03 , 0.55)		0.22	0.373	(-0.26 , 0.69)
Richest (q5)		0.53	**0.003***	(0.18 , 0.88)		0.92	**<0.001***	(0.62 , 1.22)		0.18	0.24	(-0.12 , 0.47)		0.60	**0.013***	(0.12 , 1.07)

** statistically significant at p<0.05*

Note: models adjusted for starting coverage values in each country

Table A3: Annual percentage points change in coverage (coeff) of each of the four components of the continuum of care for RMNCH comparing the periods 2000-2008 to 2008-2017 (the models do not control for starting coverage values).

**Category**		**Reproductive Health**				**Maternal health**				**Child immunization**				**Child illness treatment**			
		n	Coeff	signif	95%CI	n	Coeff	signif	95%CI	n	Coeff	signif	95%CI	n	Coeff	signif	95%CI
All countries (56)	2000-2008	193	1.28	0.00	(0.75 , 1.82)	195	1.60	0.00	(1.09 , 2.12)	200	1.32	0.00	(0.91 , 1.74)	205	1.47	0.00	(1.01 , 1.93)
	2008-2017		0.92	0.00	(0.52 1.32)		1.41	0.00	1.01 1.80		0.27	0.09	(-0.04 0.58)		0.37	0.03	0.03 0.71
Low income countries (19)	2000-2008	64	1.71	0.00	(0.58 , 2.83)	66	0.88	0.07	(-0.06 , 1.82)	68	1.42	0.00	(0.75 , 2.10)	71	1.96	0.00	(1.24 , 2.68)
	2008-2017		1.36	0.01	(0.39 2.34)		2.11	0.00	1.29 2.93		0.64	0.03	0.06 1.23		0.14	0.66	-0.47 0.75
Lower middle income countries (23)	2000-2008	79	0.57	0.15	(-0.20 , 1.33)	77	2.27	0.00	(1.34 , 3.21)	86	1.49	0.00	(0.80 , 2.17)	87	1.07	0.02	(0.20 , 1.93)
	2008-2017		1.28	0.00	(0.79 1.78)		1.58	0.00	0.96 2.19		0.34	0.14	-0.11 0.79		0.44	0.13	-0.13 1.01
Upper middle income countries (13)	2000-2008	50	0.86	0.01	(0.24 , 1.48)	52	1.81	0.00	(1.26 , 2.36)	46	0.72	0.04	(0.03 , 1.41)	47	1.31	0.00	(0.42 , 2.20)
	2008-2017		0.11	0.62	(-0.33 0.56)		0.50	0.02	0.08 0.92		-0.23	0.36	-0.73 0.26		0.55	0.08	-0.06 1.16
Education		570				571				611				605			
None	2000-2008		1.14	0.00	(0.39 , 1.90)		1.60	0.00	(0.91 , 2.29)		1.86	0.00	(1.26 , 2.46)		1.03	0.00	(0.36 , 1.70)
	2008-2017		1.24	0.00	(0.66 1.82)		1.38	0.00	0.85 1.91		-0.04	0.85	-0.50 0.42		1.17	0.00	0.66 1.69
Primary	2000-2008		1.84	0.00	(1.08 , 2.59)		1.77	0.00	(1.09 , 2.45)		1.22	0.00	(0.63 , 1.81)		1.45	0.00	(0.79 , 2.11)
	2008-2017		0.77	0.01	(0.20 1.35)		1.20	0.00	0.67 1.73		0.13	0.58	-0.32 0.58		-0.02	0.93	-0.52 0.47
Secondary or more	2000-2008		0.59	0.12	(-0.16 , 1.34)		0.46	0.18	(-0.21 , 1.14)		0.67	0.03	(0.08 , 1.25)		0.88	0.01	(0.24 , 1.53)
	2008-2017		0.42	0.15	(-0.16 0.99)		0.71	0.01	0.18 1.23		0.10	0.65	-0.35 0.56		0.00	1.00	-0.49 0.49
Place of residence		577				556				598				601			
Capital city/region	2000-2008		0.68	0.03	(0.08 , 1.29)		0.26	0.48	(-0.45 , 0.96)		0.63	0.03	(0.05 , 1.21)		0.94	0.01	(0.23 , 1.65)
	2008-2017		0.60	0.01	(0.14 1.06)		0.81	0.00	0.26 1.36		0.28	0.22	-0.17 0.73		0.53	0.06	-0.01 1.07
Other urban	2000-2008		0.76	0.01	(0.16 , 1.37)		1.06	0.00	(0.35 , 1.77)		1.06	0.00	(0.47 , 1.64)		1.49	0.00	(0.78 , 2.20)
	2008-2017		0.72	0.00	(0.26 1.19)		1.06	0.00	0.51 1.61		0.07	0.76	-0.38 0.52		-0.17	0.55	-0.71 0.38
Rural	2000-2008		1.49	0.00	(0.89 , 2.10)		2.02	0.00	(1.31 , 2.72)		1.54	0.00	(0.96 , 2.13)		1.71	0.00	(1.00 , 2.42)
	2008-2017		0.96	0.00	(0.50 1.42)		1.50	0.00	0.95 2.04		0.27	0.23	-0.17 0.72		0.20	0.47	-0.34 0.74
Maternal age		561				147				366				368			
15-19	2000-2008		1.81	0.00	(1.07 , 2.55)		1.55	0.00	(1.00 , 2.10)		1.41	0.00	(0.90 , 1.92)		2.11	0.00	(1.46 , 2.77)
	2008-2017		0.39	0.18	(-0.18 0.95)		1.55	0.00	1.00 2.10		0.22	0.32	-0.21 0.65		-0.58	0.04	-1.13 -0.02
20-34	2000-2008		1.34	0.00	(0.60 , 2.08)		1.92	0.00	(1.37 , 2.47)		1.49	0.00	(0.98 , 2.00)		1.48	0.00	(0.83 , 2.13)
	2008-2017		0.94	0.00	(0.38 1.51)		1.92	0.00	1.37 2.47		0.28	0.21	-0.15 0.71		0.52	0.06	-0.01 1.06
35-49	2000-2008		1.12	0.00	(0.38 , 1.87)		2.10	0.00	(1.55 , 2.65)		1.67	0.00	(1.16 , 2.18)		1.74	0.00	(1.09 , 2.39)
	2008-2017		1.10	0.00	(0.54 1.67)		2.10	0.00	1.55 2.65		0.63	0.00	0.20 1.06		0.60	0.03	0.07 1.14
Wealth quintile		570				585				627				630			
Poorest (q1)	2000-2008		1.31	0.00	(0.67 , 1.96)		1.72	0.00	(0.97 , 2.47)		1.36	0.00	(0.76 , 1.95)		1.56	0.00	(0.95 , 2.18)
	2008-2017		1.06	0.00	(0.57 1.56)		1.45	0.00	0.87 2.03		0.28	0.23	-0.17 0.74		0.35	0.14	-0.12 0.81
Middle (q2-q4)	2000-2008		1.36	0.00	(0.71 , 2.00)		1.76	0.00	(1.01 , 2.51)		1.51	0.00	(0.91 , 2.11)		1.57	0.00	(0.95 , 2.18)
	2008-2017		1.30	0.00	(0.81 1.80)		1.63	0.00	1.05 2.21		0.27	0.25	-0.19 0.73		0.28	0.25	-0.19 0.74
Richest (q5)	2000-2008		0.88	0.01	(0.24 , 1.53)		0.77	0.04	(0.02 , 1.52)		0.86	0.00	(0.27 , 1.46)		0.78	0.01	(0.17 , 1.40)
	2008-2017		0.52	0.04	(0.02 1.01)		0.85	0.00	0.27 1.43		0.15	0.51	-0.30 0.61		0.43	0.07	-0.04 0.89

** statistically significant at p<0.05*

Note: the models do not control for starting coverage values and therefore the coefficients for the period 2008-2017 are slightly different from the those in the table A2

Amouzou A, *et al. BMJ Global Health* 2020; 5:e002230. doi: 10.1136/bmjgh-2019-002230

Figure A2: Comparison between linear regression of coverage indicators and its logic transformed

A. Predicted coverage estimate using multilevel linear regression of coverage

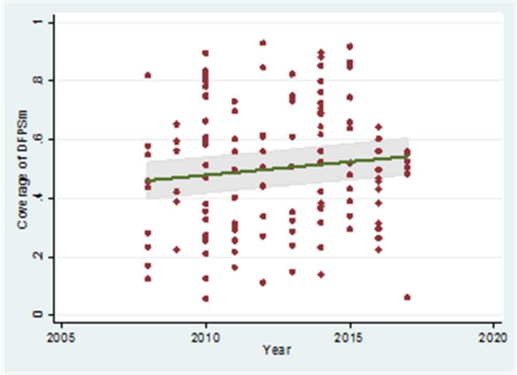

B. Predicted coverage estimate using multilevel linear regression of logit transformed of coverage

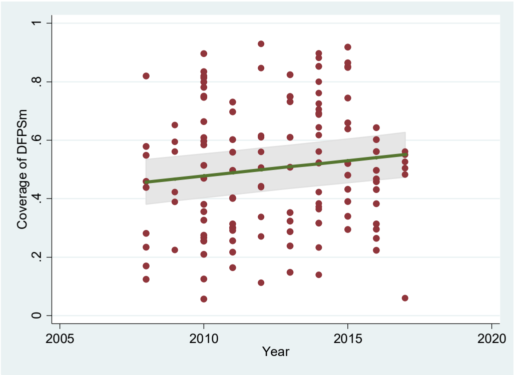

FIGURE A3 - SCATTERPLOTS WITH PREDICTIONS ON THE PERIOD 2008-2017

REPRODUCTIVE HEALTH

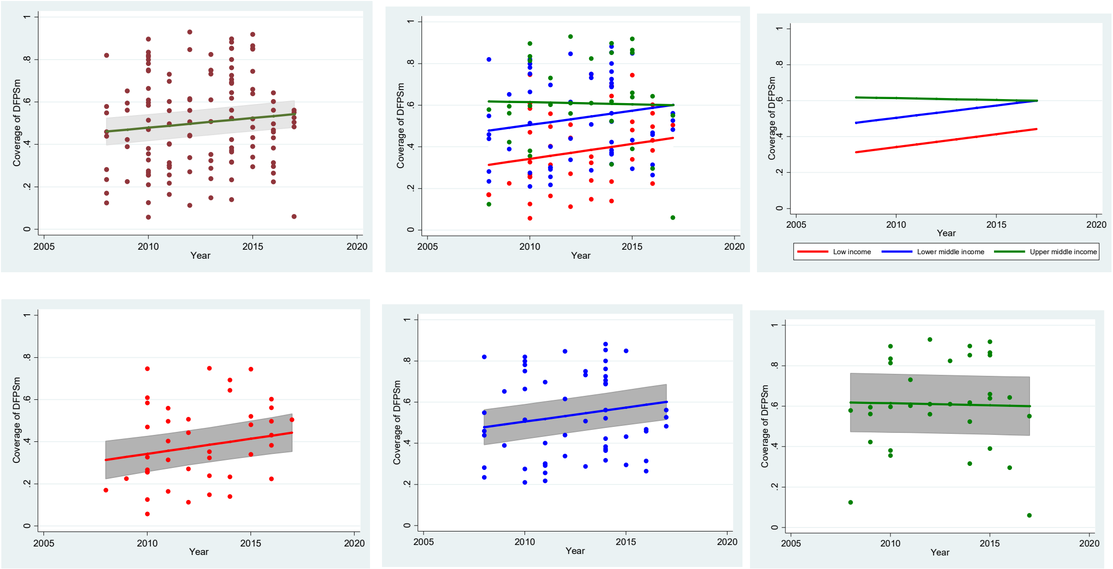

MATERNAL HEALTH

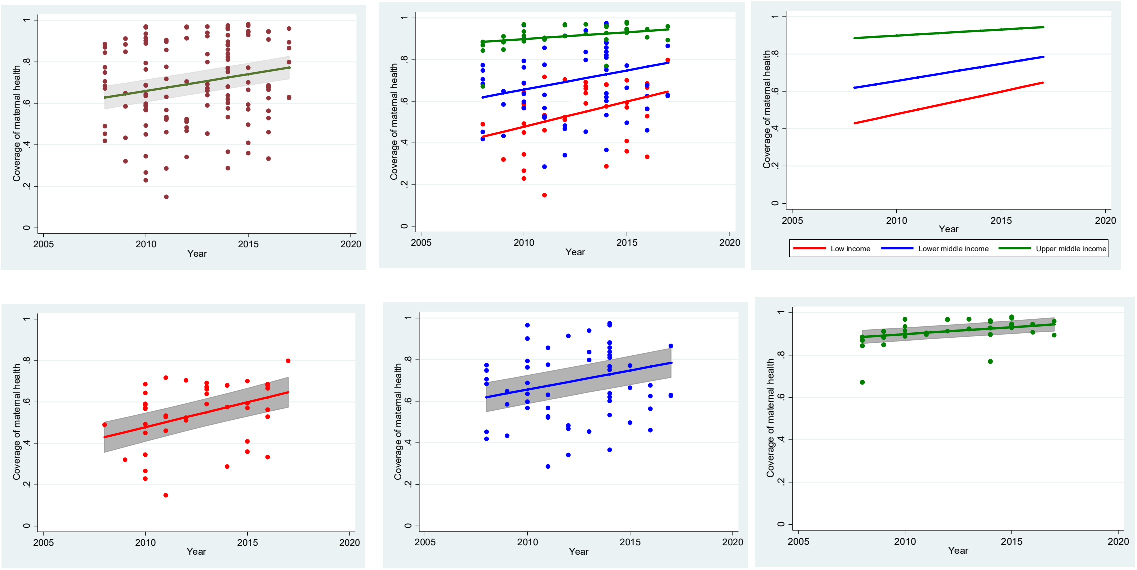

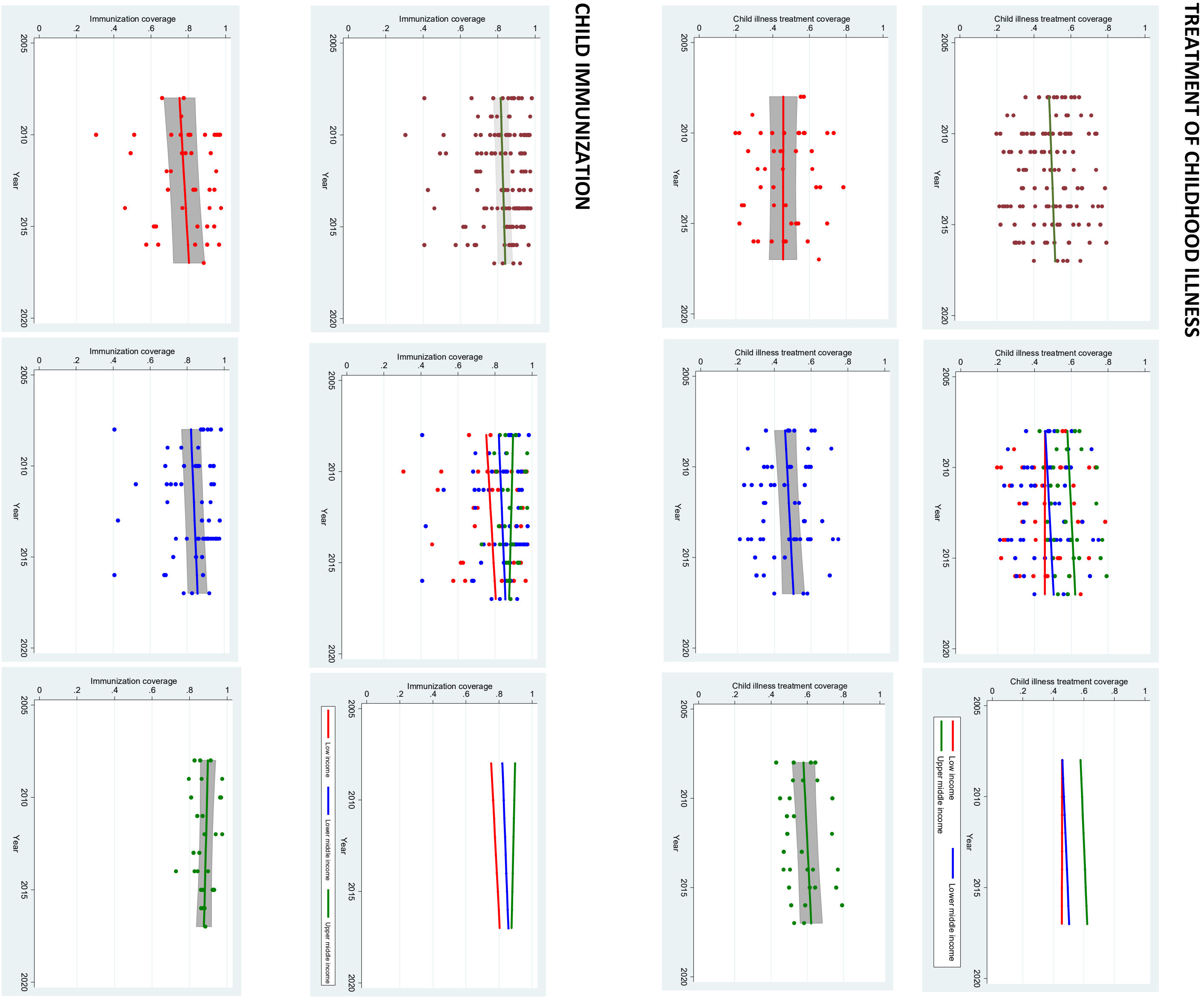
CHILD IMMUNIZATION
TREATMENT OF CHILDHOOD ILLNESS
Immunization coverage
Child illness treatment coverage
Year
Low income
Lower middle income
Upper middle income

FIGURE A4 – SCATTERPLOT SHOWING TRENDS SINCE 2000 WITH SPLINE KNOT AT 2008 TO DISTINGUISHED TREND ON PERIOD 2000-2008 AND 2008-2017

REPRODUCTIVE HEALTH

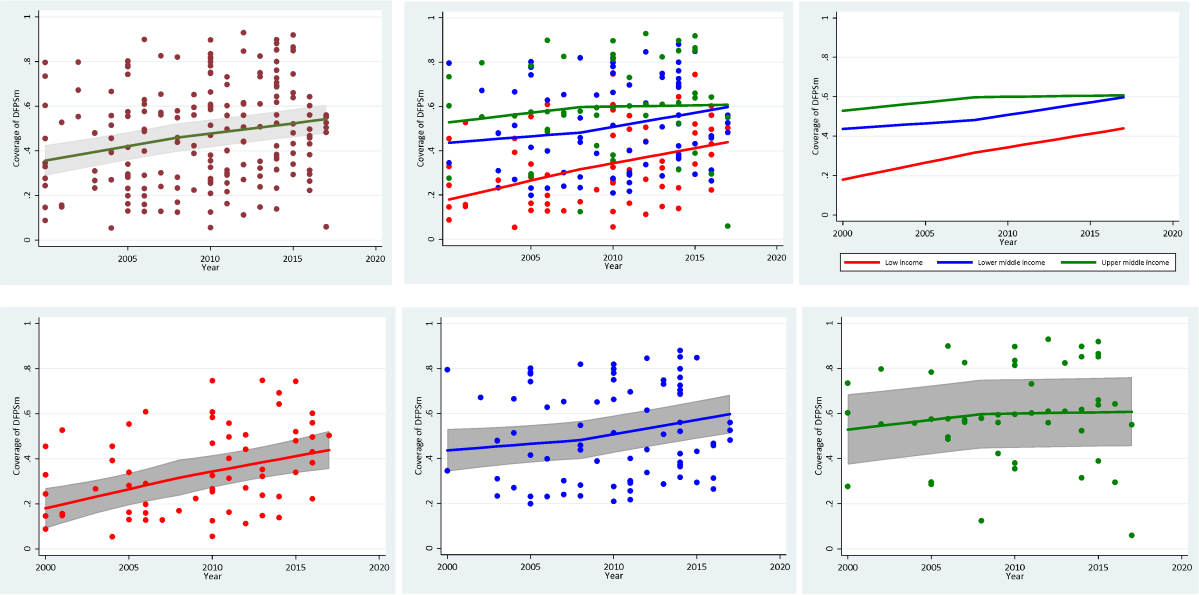

MATERNAL HEALTH

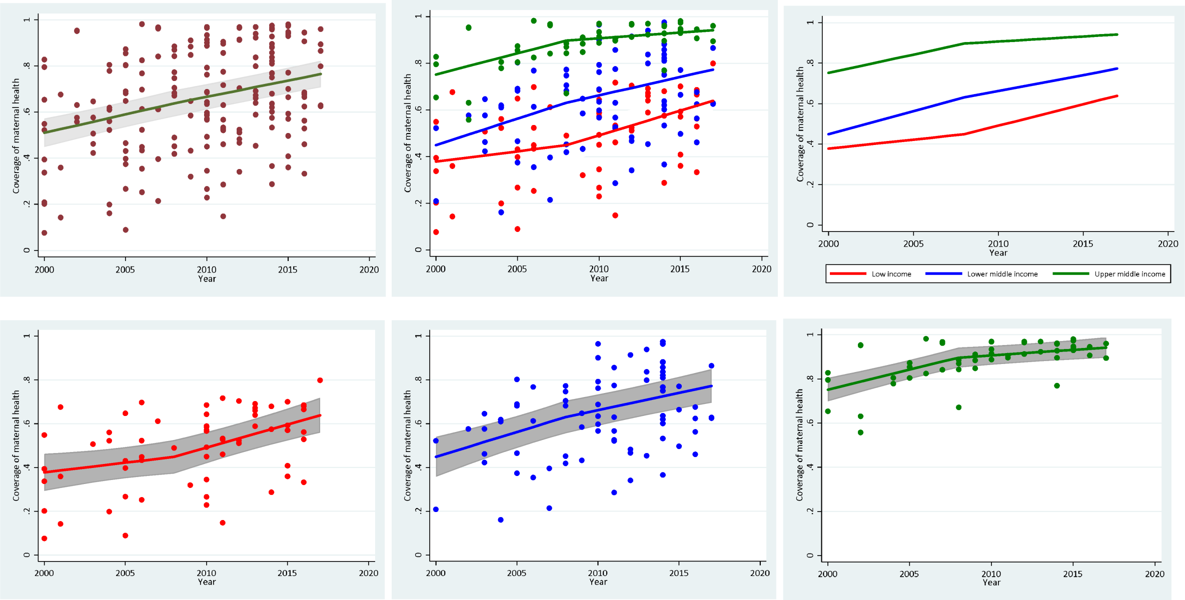

CHILD IMMUNIZATION

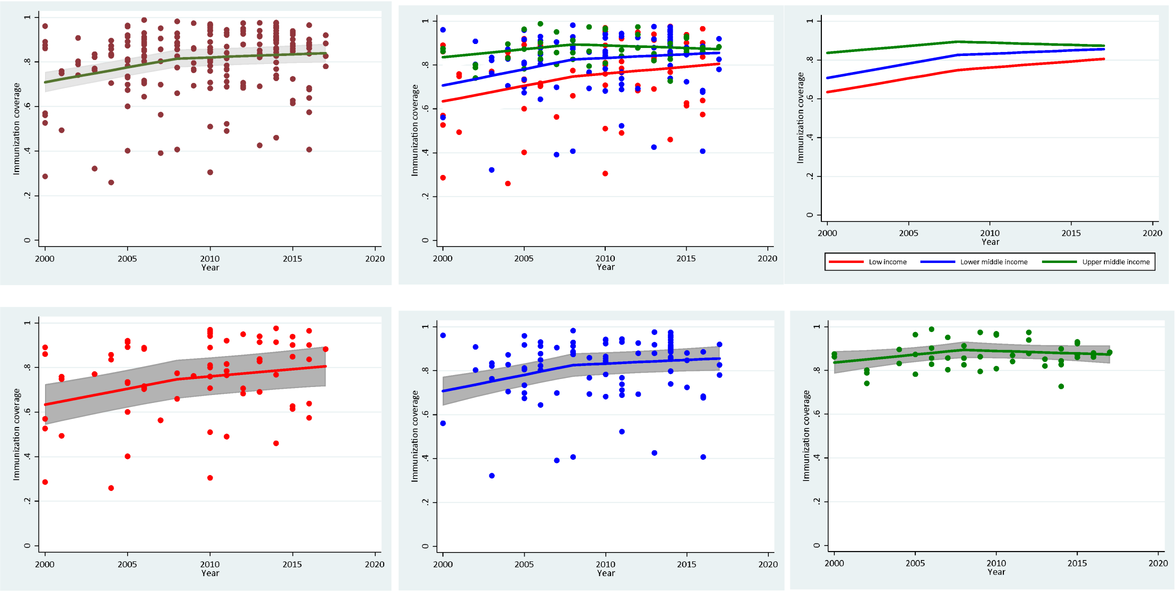

CHILD ILLNESS TREATMENT

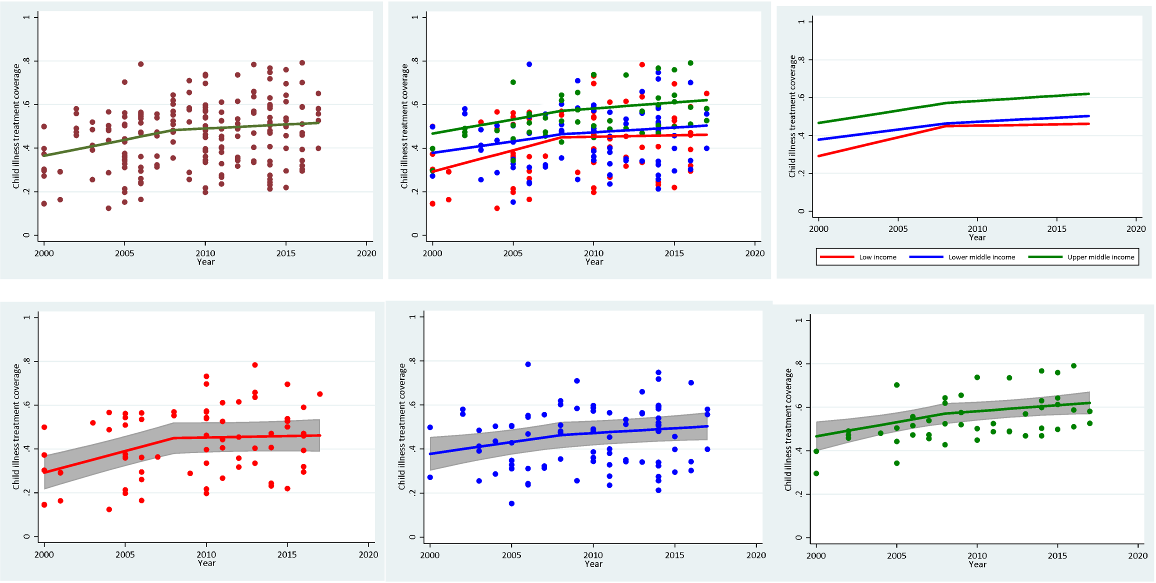

Table A4 – Reproductive Health: Country specific average percentage point change in coverage during the period 2008-2017

country	iso	year_start	source_st	year_end	source_en	Poorest_q	Middle_2_4_	Richest_q	No_Education	Primary_Educ	Secondary_or	15_19_years_o	20_34_years_o	35_49_years_o	Capital_city	Other_urban	Rural
Albania	ALB	2008	DHS	2017.00	DHS	-0.67	-0.61	-1.13	1.14	-0.57	-0.96	-1.29	-0.99	-0.55	-0.86	-0.78	-0.62
Armenia	ARM	2010	DHS	2015.00	DHS	-0.30	0.25	0.09		-14.49	0.20	6.75	0.27	-0.11	0.28	-0.28	0.79
Burundi	BDI	2010	DHS	2016.00	DHS	1.52	1.02	0.30	1.10	0.86	-0.63	4.45	1.09	0.66	-0.29	0.60	1.03
Benin	BEN	2011	DHS	2014.00	MICS	1.65	1.75	4.52	2.00	1.47	1.90				2.23	3.17	1.84
Banglades	BGD	2011	DHS	2014.00	DHS	0.86	1.13	0.57	0.66	1.49	0.72	0.77	1.01	0.86	0.69	0.94	0.98
Belize	BLZ	2011	MICS	2015.00	MICS	-1.89	-1.78	-1.70	-7.03	-1.10	-2.35	-1.14	-1.64	-1.55	-2.33	-2.57	-1.22
Cote_dIvoi	CIV	2011	DHS	2016.00	MICS	1.24	0.94	1.89	0.93	0.95	1.57	0.24	1.17	1.20	0.40	2.12	0.81
Cameroon	CMR	2011	DHS	2014.00	MICS	0.52	2.21	3.99	0.35	3.05	2.79	0.47	2.21	2.26	4.44	2.63	1.24
Congo_De	COD	2010	MICS	2013.00	DHS	1.00	1.03	0.15	1.24	0.08	0.73	0.89	0.84	0.53	1.37	1.39	0.14
Congo_Bra	COG	2011	DHS	2014.00	MICS	2.81	3.33	1.31	2.72	2.37	1.90	-1.00	2.79	3.39	4.36	-0.77	3.60
Colombia	COL	2010	DHS	2015.00	DHS	0.83	0.71	0.04	0.22	0.51	0.60	1.45	0.69	0.40	0.88	0.61	0.37
Cuba	CUB	2010	MICS	2014.00	MICS					-0.07	0.02	-3.20	-0.08	0.17	-0.52	0.56	-0.85
Dominican	DOM	2013	DHS	2014.00	MICS	0.13	3.42	3.22	0.72	1.32	3.87	7.10	3.10	1.02	2.13	4.13	1.13
Egypt	EGY	2008	DHS	2014.00	DHS	0.16	-0.32	-0.75	-0.17	-0.50	-0.41	0.30	0.00	-0.76	-0.82	-0.71	-0.01
Ethiopia	ETH	2011	DHS	2016.00	DHS	2.63	2.63	0.49	2.29	1.99	0.11	4.00	2.08	1.70	-0.52	1.52	2.63
Ghana	GHA	2008	DHS	2014.00	DHS	2.82	2.08	-0.39	2.26	2.91	0.93	2.32	2.31	0.72	-0.97	1.41	2.59
Guinea	GIN	2012	DHS	2016.00	MICS	0.88	2.86	3.72	2.76	0.99	2.47	0.79	3.20	3.52	3.32	1.87	2.33
Gambia	GMB	2010	MICS	2013.00	DHS	0.26	-0.59	-2.25	-0.60	-6.71	-0.28	0.36	-0.59	-0.76	-1.67	-0.95	-0.51
Guyana	GUY	2009	DHS	2014.00	MICS	0.26	-1.00	-0.87	2.93	0.10	-1.04	-5.86	-0.84	0.74	-1.88	-1.89	-0.27
Haiti	HTI	2012	DHS	2016.00	DHS	-1.64	-0.15	0.65	-0.66	-0.02	-0.42	-0.04	-0.68	0.10	1.56	-0.90	-0.79
Jordan	JOR	2012	DHS	2017.00	DHS	-0.36	-0.36	0.69	-1.40	-0.45	-0.15	-4.43	-0.43	0.07	0.19	-0.52	-0.38
Kazakhsta	KAZ	2010	MICS	2015.00	MICS	2.14	0.66	-0.09		5.35	0.76	3.00	1.20	0.33	-3.07	0.57	1.36
Kenya	KEN	2008	DHS	2014.00	DHS	2.81	2.58	2.40	1.13	3.14	1.62	3.17	3.03	1.82	2.38	2.36	2.36
Kyrgyzsta	KGZ	2012	DHS	2014.00	MICS	0.76	4.04	4.73	-33.31	-21.41	3.66	-0.88	2.09	6.78	5.54	3.52	3.23
Cambodia	KHM	2010	DHS	2014.00	DHS	0.94	1.51	0.50	1.11	1.25	1.30	0.61	1.15	1.02	-0.52	0.72	1.33
Lesotho	LSO	2009	DHS	2014.00	DHS	4.77	2.46	0.21	0.52	2.79	1.28	1.68	2.13	2.36	0.32	1.41	2.77
Maldives	MDV	2009	DHS	2016.00	DHS	-2.25	-2.02	-0.69	-2.20	-1.22	-0.91	-1.03	-1.39	-2.56	-1.49		-2.04
Mali	MLI	2009	MICS	2015.00	MICS	0.54	1.95	3.41	1.88	1.72	1.57	2.00	1.11	2.10	4.10	1.88	2.05
Mongolia	MNG	2010	MICS	2013.00	MICS	-0.57	-1.80	-2.24	-2.90	1.39	-1.81	-19.27	-2.38	-0.57	-1.99	-1.99	-0.86
Mauritania	MRT	2011	MICS	2015.00	MICS	1.27	2.27	2.39	2.18	-1.47	-0.07	0.15	1.69	2.60	2.60	1.15	1.49
Malawi	MWI	2010	DHS	2015.00	DHS	3.93	3.28	2.49	3.55	3.39	1.90	2.62	3.38	2.95	3.49	2.13	3.34
Nigeria	NGA	2008	DHS	2016.00	MICS	0.16	0.51	0.74	0.75	0.61	0.26	-0.24	0.94	1.20	0.03	0.91	0.30
Nepal	NPL	2010	MICS	2016.00	DHS	-0.26	-3.07	-5.12	-1.73	-2.38	-3.69	-2.88	-3.25	-3.15	-3.91	-3.97	-3.25
Pakistan	PAK	2012	DHS	2017.00	DHS	1.58	0.91	0.06	0.69	1.94	0.22	0.02	1.00	0.86	-1.06	-0.17	1.41
Peru	PER	2008	DHS	2016.00	DHS	1.55	1.15	0.69	0.01	0.70	0.62	1.31	1.09	0.42	0.77	0.37	1.04
Philippines	PHL	2008	DHS	2017.00	DHS	2.43	0.95	0.32	2.11	1.64	0.96	2.40	1.38	0.79	1.62	0.36	1.44
State_of_F	PSE	2010	MICS	2014.00	MICS	1.46	0.53	0.35	2.85	1.04	0.73	1.26	0.80	0.30	1.50	0.51	0.82
Rwanda	RWA	2010	DHS	2014.00	DHS	2.27	0.65	0.30	1.04	0.93	-0.14	2.33	0.72	1.26	0.84	0.57	0.88
Sudan	SDN	2010	MICS	2014.00	MICS	2.11	2.67	3.14	1.53	2.43	2.38	1.01	2.55	3.00	3.32	2.64	2.92
Senegal	SEN	2010	DHS	2017.00	DHS	3.52	3.83	3.29	3.75	3.60	1.81	1.62	3.73	3.46	4.90	3.19	3.39
Sierra_Leo	SLE	2008	DHS	2013.00	DHS	4.17	4.05	2.46	3.91	4.07	1.97	2.19	4.40	2.89	1.48	4.75	4.01
Serbia	SRB	2010	MICS	2014.00	MICS	-0.67	-0.89	-1.71	7.34	-0.87	-1.08	-6.67	-0.41	-1.15	2.42	-2.10	-1.32
Sao_Tome	STP	2008	DHS	2014.00	MICS	1.68	1.59	0.53	2.34	0.67	2.59	1.57	1.09	1.88	4.35	0.31	1.03
Eswatini	SWZ	2010	MICS	2014.00	MICS	2.30	0.55	0.58	1.53	1.05	0.45	-0.65	0.82	0.83	2.12	0.05	0.89
Chad	TCD	2010	MICS	2014.00	DHS	1.94	2.10	2.16	1.48	1.93	3.65	0.52	1.81	3.25	0.64	4.16	2.06
Togo	TGO	2010	MICS	2013.00	DHS	3.14	2.60	0.21	2.30	2.30	1.42	2.25	2.36	2.01	3.49	0.21	2.76
Thailand	THA	2012	MICS	2015.00	MICS	0.13	-0.69	0.32	-1.76	-0.23	0.24	1.03	0.24	-0.78	0.47	-0.29	-0.52
Tajikistan	TJK	2012	DHS	2017.00	DHS	0.51	0.11	-0.83	0.50	0.00	-0.05	-0.06	0.24	-0.39	1.35	-0.67	-0.08
Timor_Les	TLS	2009	DHS	2016.00	DHS	2.38	1.38	-0.78	2.24	1.18	0.23	0.02	0.81	1.39	-1.05	-0.05	1.71
Tanzania	TZA	2010	DHS	2015.00	DHS	0.36	2.02	-0.87	2.19	0.89	-0.40	-0.63	1.38	0.77	-0.83	0.22	1.48
Uganda	UGA	2011	DHS	2016.00	DHS	2.43	2.42	0.34	1.82	2.28	0.36	1.95	2.13	1.48	-1.51	0.96	2.15
Vietnam	VNM	2010	MICS	2013.00	MICS	-0.22	-0.89	-0.25	0.29	-0.66	-0.71	6.52	-0.63	-0.67	-2.53	-0.58	-0.45

Figure A5- Average annual percentage points change in reproductive health coverage by country and equity group

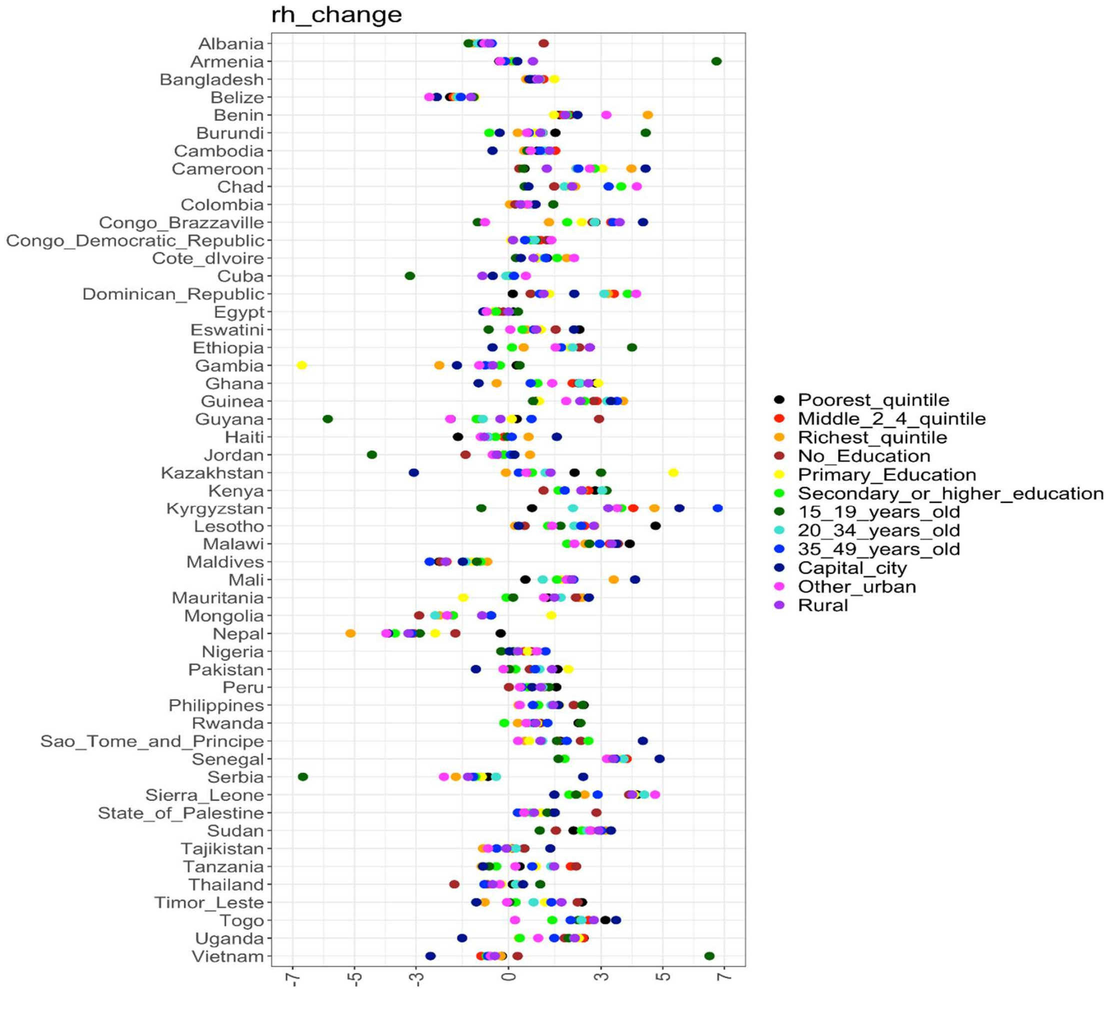

Table A5 – Maternal Health: Country specific average percentage point change in coverage during the period 2008-2017

country	iso	year_start	source_start	year_end	source_end	Poorest_quintile	Middle_2_4_quintile	Richest_quintile	No_Education	Primary_Education	Secondary_or_higher_education	15_19_years_old	20_34_years_old	35_49_years_old	Capital_city	Other_urban	Rural
Afghanistan	AFG	2010	MICS	2015.00	DHS	1.59	2.09	1.54	1.69	1.00	-0.41						
Albania	ALB	2008	DHS	2017.00	DHS	1.02	0.51	0.12	1.30	0.47	0.25				-0.35	-0.02	0.86
Armenia	ARM	2010	DHS	2015.00	DHS	0.22	0.32	-0.03			0.26	-0.02	0.25	0.37	0.07	0.20	0.42
Burundi	BDI	2010	DHS	2016.00	DHS	3.20	3.46	2.47	3.34	3.09	1.32	3.23	3.19	3.26	0.81	2.29	3.35
Benin	BEN	2011	DHS	2014.00	MICS	-1.42	-1.24	-0.31	-1.50	-2.92	-2.06				2.00	-1.49	-1.19
Bangladesh	BGD	2011	DHS	2014.00	DHS	1.88	2.79	2.09	1.62	2.31	2.24	2.44	2.79	2.74	1.01	1.48	2.78
Belize	BLZ	2011	MICS	2015.00	MICS	2.98	0.63	0.87	6.34	0.98	1.10				1.67	0.34	1.59
Cote_dIvoire	CIV	2011	DHS	2016.00	MICS	1.73	2.59	1.52	2.33	0.82	1.23				0.58	1.89	2.31
Cameroon	CMR	2011	DHS	2014.00	MICS	0.94	-0.64	0.69	1.31	-1.17	-0.46				0.38	0.16	-0.24
Congo_Democratic_Repul	COD	2010	MICS	2013.00	DHS	1.09	1.89	1.07	1.93	0.81	1.10				0.59	1.15	1.54
Congo_Brazzaville	COG	2011	DHS	2014.00	MICS	-1.39	0.90	0.00	-0.99	-0.37	-0.53				1.40	0.33	-1.36
Colombia	COL	2010	DHS	2015.00	DHS	0.65	0.26	-0.02	-2.93	0.49	0.10	0.15	0.35	0.29	-0.35	0.49	0.38
Dominican_Republic	DOM	2013	DHS	2014.00	MICS	0.83	-1.74	-1.26	4.72	-1.59	-1.18				-0.20	-2.09	-0.89
Egypt	EGY	2008	DHS	2014.00	DHS	4.69	2.07	0.35	3.34	2.48	1.50				1.63	0.76	3.14
Ethiopia	ETH	2011	DHS	2016.00	DHS	2.23	4.13	4.43	2.98	4.10	1.44	4.30	3.69	3.24	1.69	5.62	3.65
Ghana	GHA	2008	DHS	2014.00	DHS	3.46	1.65	0.45	2.51	1.91	1.22				1.02	0.79	2.54
Guinea	GIN	2012	DHS	2016.00	MICS	-0.02	1.96	-0.62	0.90	0.36	0.15				-0.18	0.66	0.72
Gambia	GMB	2010	MICS	2013.00	DHS	2.76	0.64	-0.53	0.96	-0.25	-0.54				-0.45	0.14	1.27
Guatemala	GTM	2008	RHS	2014.00	DHS	2.16	1.29	-0.07	1.98	1.18	0.19						
Guyana	GUY	2009	DHS	2014.00	MICS	0.84	1.07	0.97	1.36	0.76	0.77				0.15	1.54	1.01
Haiti	HTI	2012	DHS	2016.00	DHS	0.09	0.50	-0.03	-0.31	-0.18	-0.32	-0.34	-0.04	1.07	-1.17	1.37	0.35
Jordan	JOR	2012	DHS	2017.00	DHS	0.27	-0.27	-0.26	-1.47	-0.06	-0.16	0.10	-0.24	-0.02	-0.20	-0.23	-0.06
Kazakhstan	KAZ	2010	MICS	2015.00	MICS	0.62	0.64	1.46							3.62	0.85	0.49
Kenya	KEN	2008	DHS	2014.00	DHS	1.63	2.96	1.84	1.30	2.52	1.49				0.33	1.84	1.95
Kyrgyzstan	KGZ	2012	DHS	2014.00	MICS	1.88	3.54	-0.29	-31.83	13.72	2.65				0.81	1.06	3.24
Cambodia	KHM	2010	DHS	2014.00	DHS	4.85	3.75	0.81	4.33	3.82	1.44	2.52	3.60	4.47	0.08	1.45	4.30
Lesotho	LSO	2009	DHS	2014.00	DHS	3.79	2.42	0.50	1.03	2.69	1.07				-0.88	0.14	3.14
Maldives	MDV	2009	DHS	2016.00	DHS	-0.01	-0.18	0.30	0.67	0.35	-0.37				0.12		-0.16
Mali	MLI	2009	MICS	2015.00	MICS	0.97	1.62	0.59	1.26	1.67	-1.22				0.96	2.07	1.84
Mongolia	MNG	2010	MICS	2013.00	MICS	0.73	1.22	1.98	0.60	1.06	1.38				1.86	0.87	0.86
Mozambique	MOZ	2008	MICS	2015.00	DHS							1.16	1.73	1.47			
Mauritania	MRT	2011	MICS	2015.00	MICS	2.00	2.61	2.44	3.69	-1.48	-1.92				3.46	1.87	1.84
Malawi	MWI	2010	DHS	2015.00	DHS	2.99	2.29	1.47	2.55	2.32	1.25	1.42	2.45	2.61	2.84	1.72	2.33
Nigeria	NGA	2008	DHS	2016.00	MICS	0.62	0.52	0.10	1.07	0.12	-0.28				0.15	0.34	0.67
Nepal	NPL	2010	MICS	2016.00	DHS	6.09	5.40	3.07	4.25	3.24	4.10	3.89	3.94	4.48	0.43	3.60	4.32
Pakistan	PAK	2012	DHS	2017.00	DHS	2.47	3.59	1.40	2.90	3.03	1.84	2.39	3.27	3.01	-1.08	2.20	3.36
Peru	PER	2008	DHS	2016.00	DHS	2.43	1.18	0.19	1.56	1.19	0.39	0.71	0.86	1.43	0.31	0.28	1.53
Philippines	PHL	2008	DHS	2017.00	DHS	3.08	1.61	0.40	2.59	2.16	1.39	0.20	1.79	2.00	1.09	1.10	2.50
Paraguay	PRY	2008	RHS	2016.00	MICS	1.26	0.46	0.34	1.44	0.83	0.27						
State_of_Palestine	PSE	2010	MICS	2014.00	MICS	0.76	0.07	0.25	2.48	0.11	0.08				-0.12	0.21	0.76
Rwanda	RWA	2010	DHS	2014.00	DHS	2.89	3.34	0.83	2.74	2.81	1.38	0.82	2.87	3.38	1.32	2.33	2.93
Sudan	SDN	2010	MICS	2014.00	MICS	-0.06	1.11	1.18	0.59	0.58	0.12				1.41	0.88	1.10
Senegal	SEN	2010	DHS	2017.00	DHS	1.10	0.87	0.66	0.57	0.31	0.57	0.81	1.01	0.46	0.69	0.21	1.28
Sierra_Leone	SLE	2008	DHS	2013.00	DHS	5.01	4.12	2.40	4.28	2.81	2.18				1.52	3.97	4.54
Serbia	SRB	2010	MICS	2014.00	MICS	-0.44	-0.08	-0.70	-5.12	0.34	-0.25				-1.49	0.23	-0.05
Sao_Tome_and_Principe	STP	2008	DHS	2014.00	MICS	1.97	2.11	0.62	2.81	1.62	1.27				0.82	1.91	2.11
Eswatini	SWZ	2010	MICS	2014.00	MICS	1.72	0.58	0.20	1.09	1.30	0.24				2.65	0.39	0.63
Chad	TCD	2010	MICS	2014.00	DHS	2.46	1.42	1.50	1.16	1.14	0.19				1.55	1.62	1.94
Togo	TGO	2010	MICS	2013.00	DHS	0.96	0.44	0.07	1.44	-0.54	-0.96				0.04	-0.75	0.27
Thailand	THA	2012	MICS	2015.00	MICS	0.08	-0.94	-0.15	-5.78	-1.54	-0.35				0.24	-0.72	-0.83
Tajikistan	TJK	2012	DHS	2017.00	DHS	3.26	1.58	1.27	2.58	1.75	1.87	1.08	1.76	4.14	2.51	1.68	1.94
Timor_Leste	TLS	2009	DHS	2016.00	DHS	2.64	3.78	3.09	2.65	2.89	2.93				3.30	4.54	3.12
Tanzania	TZA	2010	DHS	2015.00	DHS	1.53	2.69	2.10	1.57	2.06	0.51	2.59	2.36	2.42	1.21	1.79	2.13
Uganda	UGA	2011	DHS	2016.00	DHS	3.29	3.22	1.42	3.23	2.82	2.17	2.19	3.18	2.44	0.47	1.81	3.02
Vietnam	VNM	2010	MICS	2013.00	MICS	2.14	2.92	1.34	0.81	3.32	2.30				0.70	0.90	3.19
Zimbabwe	ZWE	2009	MICS	2015.00	DHS	3.79	3.01	2.08	5.54	3.91	2.55	3.22	2.58	2.88	1.27	1.85	3.65

Figure A6- Average annual percentage points change in maternal health coverage by country and equity group

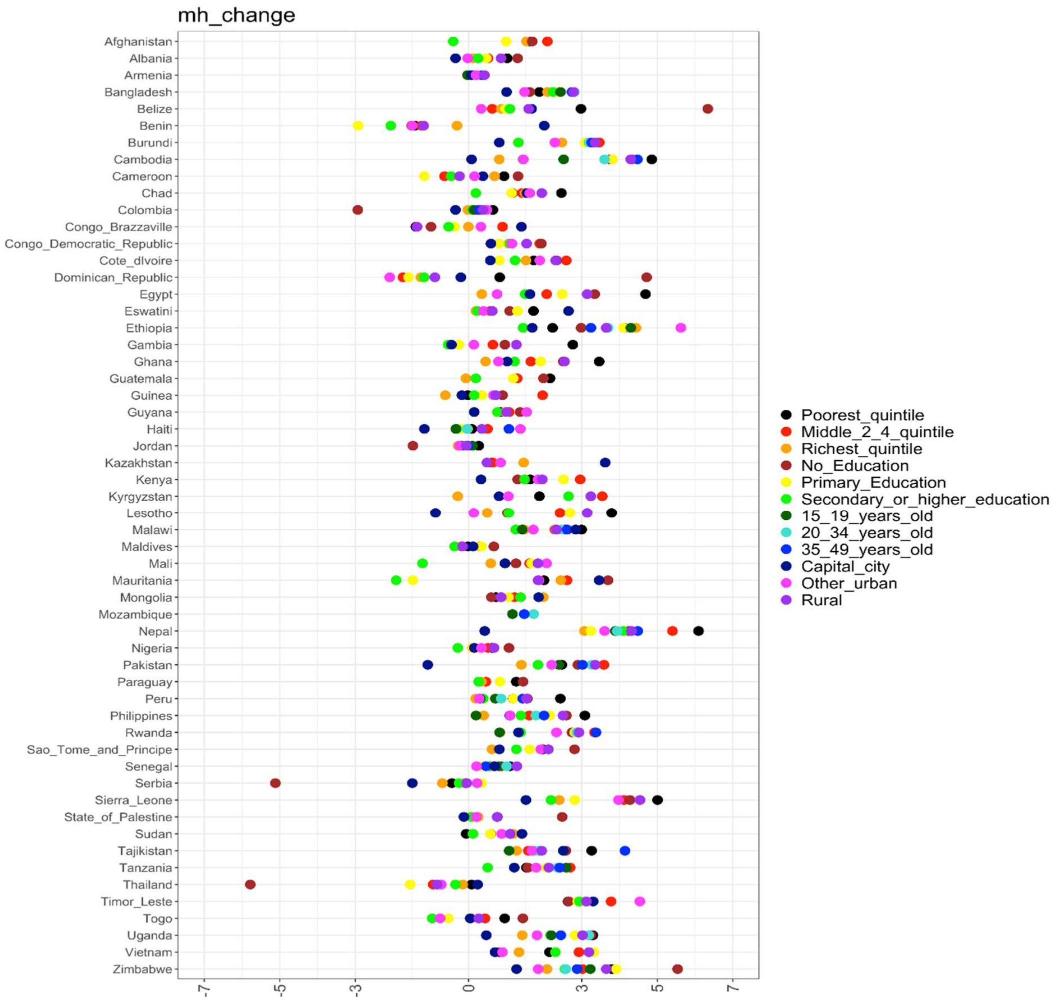

Table A6 – Child Immunization: Country specific average percentage point change in coverage during the period 2008-2017

country	iso	year_start	source_start	year_end	source_end	Poorest_quintile	Middle_2_4_quintile	Richest_quintile	No_Education	Primary_Education	Secondary_or_higher_education	15_19_years_old	20_34_years_old	35_49_years_old	Capital_city	Other_urban	Rural
Afghanistan	AFG	2010	MICS	2015	DHS	2.88	2.20	1.89	1.96	1.51	1.78						
Armenia	ARM	2010	DHS	2015	DHS	0.79	-1.17	-0.76			-0.71	0.00	-0.68	-0.88	-0.98	-0.90	-0.39
Burundi	BDI	2010	DHS	2016	DHS	0.06	0.05	0.07	-0.01	0.08	0.17	0.28	0.11	-0.14	0.13	0.27	0.04
Benin	BEN	2011	DHS	2014	MICS	0.60	-0.12	0.91	0.11	-0.90	-1.52				0.17	-0.73	0.73
Bangladesh	BGD	2011	DHS	2014	DHS	-1.93	0.02	-0.14	-1.00	-0.79	-0.54	-0.01	-0.63	0.02	0.83	-0.50	-0.69
Belize	BLZ	2011	MICS	2015	MICS	0.27	0.73	1.57	5.53	-0.40	1.72				-1.05	2.88	0.16
Cote_dIvoire	CIV	2011	DHS	2016	MICS	-0.63	0.16	-0.30	-0.31	-0.57	-1.28				-1.71	-0.93	0.42
Cameroon	CMR	2011	DHS	2014	MICS	4.37	4.33	2.64	5.71	4.56	2.92				3.43	3.34	4.67
Congo_Democrat	COD	2010	MICS	2013	DHS	-0.07	-1.06	0.19	0.55	-1.73	-0.73				0.24	-0.68	-0.87
Congo_Brazzavill	COG	2011	DHS	2014	MICS	-2.00	-0.31	-1.43	-1.79	-1.08	-1.91				1.01	-2.97	-1.35
Dominican_Repu	DOM	2013	DHS	2014	MICS	-9.06	-10.19	-8.04	3.02	-7.81	-10.71				-9.20	-7.85	-11.54
Egypt	EGY	2008	DHS	2014	DHS	-0.46	-0.05	-0.13	-0.45	0.12	-0.12	-0.45	-0.17	0.13	-0.28	-0.07	-0.11
Ethiopia	ETH	2011	DHS	2016	DHS	0.91	2.34	1.07	1.53	1.58	-0.42	-0.14	1.86	1.46	0.49	1.91	1.80
Ghana	GHA	2008	DHS	2014	DHS	0.14	-0.08	-0.18	0.22	-0.67	-0.01	0.99	-0.15	0.08	-0.10	-0.17	0.04
Gambia	GMB	2010	MICS	2013	DHS	-0.86	-0.85	-3.41	-0.65	-2.26	-2.46				-3.80	-1.99	-0.58
Guatemala	GTM	2008	RHS	2014	DHS	-1.40	-0.27	0.36	-1.38	-0.58	0.19						
Guyana	GUY	2009	DHS	2014	MICS	2.35	-0.37	0.59	2.00	0.76	0.63				0.33	-0.48	0.91
Haiti	HTI	2012	DHS	2016	DHS	-2.45	-1.33	2.82	-0.62	-2.54	-0.33	-2.79	-1.22	-0.23	-0.36	0.58	-1.83
Jordan	JOR	2012	DHS	2017	DHS	-1.85	-1.76	-2.11	-5.01	-2.07	-1.76	-1.59	-2.03	-1.23	-2.17	-1.60	-1.30
Kazakhstan	KAZ	2010	MICS	2015	MICS	-0.48	-0.66	-1.36							-3.11	-0.93	-0.26
Kenya	KEN	2008	DHS	2014	DHS	0.53	0.49	0.31	-0.82	0.62	0.26	1.08	0.33	0.57	0.78	0.34	0.34
Kyrgyzstan	KGZ	2012	DHS	2014	MICS	-0.34	1.55	2.95		10.51	1.55				4.39	-0.96	1.15
Cambodia	KHM	2010	DHS	2014	DHS	-0.32	-0.55	0.84	0.82	-1.18	0.14	-0.09	-0.24	-0.29	1.41	-0.19	-0.33
Lesotho	LSO	2009	DHS	2014	DHS	2.60	0.76	-0.92	-5.11	1.47	0.27	0.52	0.45	3.30	-1.61	-0.08	1.36
Maldives	MDV	2009	DHS	2016	DHS	-1.46	-1.17	-1.92	-0.05	-1.66	-1.31	-7.68	-1.40	-1.12	-1.09		-1.48
Mali	MLI	2009	MICS	2015	MICS	-3.10	-2.58	-1.74	-2.69	-2.54	-3.12				-0.51	-4.42	-2.44
Mongolia	MNG	2010	MICS	2013	MICS	1.34	2.06	1.13	2.59	-0.28	1.89				1.48	1.72	1.99
Mozambique	MOZ	2008	MICS	2015	DHS	1.66	0.93	0.80	1.04	0.96	0.37				0.29	0.67	1.27
Mauritania	MRT	2011	MICS	2015	MICS	-0.19	-0.19	2.45	1.07	-1.67	-1.37				2.83	-0.13	-0.86
Malawi	MWI	2010	DHS	2015	DHS	0.12	-0.05	-0.23	-0.06	-0.07	-0.33	-0.01	-0.04	-0.12	-0.30	-1.03	0.05
Nigeria	NGA	2008	DHS	2016	MICS	-0.07	-0.01	-0.43	0.82	0.17	-0.97	1.73	0.19	0.54	0.90	-0.01	-0.04
Nepal	NPL	2010	MICS	2016	DHS	2.25	1.42	0.88	1.47	0.40	1.18				4.03	2.35	1.35
Pakistan	PAK	2012	DHS	2017	DHS	2.89	1.29	0.60	1.42	1.56	0.76	0.06	1.76	1.94	-2.37	0.58	2.18
Peru	PER	2008	DHS	2016	DHS	0.61	0.69	0.48	0.15	0.41	0.61	0.78	0.56	0.56	1.03	0.49	0.34
Philippines	PHL	2008	DHS	2017	DHS	-0.38	-0.52	-0.45	-0.36	-0.86	-0.67	-0.09	-0.81	0.29	-0.25	-0.56	-0.52
Paraguay	PRY	2008	RHS	2016	MICS	-0.46	-0.76	-0.61	-0.91	-1.22	-0.78						
State_of_Palestir	PSE	2010	MICS	2014	MICS	0.89	0.60	-0.24	1.63	0.39	0.64				0.31	0.54	0.79
Rwanda	RWA	2010	DHS	2014	DHS	-0.31	0.38	-0.03	-0.22	0.18	0.13	1.05	0.11	0.21	0.06	0.43	0.13
Sudan	SDN	2010	MICS	2014	MICS	2.31	3.17	0.89	2.56	1.40	2.37				2.98	2.48	3.02
Senegal	SEN	2010	DHS	2017	DHS	0.79	0.91	1.17	0.88	0.90	0.08	1.08	0.90	0.97	2.01	0.46	0.75
Sierra_Leone	SLE	2008	DHS	2013	DHS	4.48	3.54	0.78	3.76	1.86	1.36	3.47	3.35	3.30	0.00	3.07	3.89
El_Salvador	SLV	2008	RHS	2014	MICS	0.47	-0.07	-0.61	-0.90	-0.30	0.17						
Sao_Tome_and_I	STP	2008	DHS	2014	MICS	1.16	1.06	0.95	1.94	0.79	1.40				1.49	1.21	0.64
Eswatini	SWZ	2010	MICS	2014	MICS	-0.17	-0.38	1.29	-0.76	-1.03	0.49				-1.52	0.86	-0.18
Chad	TCD	2010	MICS	2014	DHS	5.43	3.79	3.60	3.45	3.43	2.49				3.44	2.28	4.43
Togo	TGO	2010	MICS	2013	DHS	6.20	1.40	1.10	2.80	2.37	1.04				1.72	1.83	3.10
Thailand	THA	2012	MICS	2015	MICS	-1.22	-0.30	-1.22	-2.26	-1.73	0.73				2.61	-1.09	-0.68
Tajikistan	TJK	2012	DHS	2017	DHS	-0.99	-1.26	-2.24	0.18	-0.71	-1.43	-0.03	-1.34	-1.37	-2.25	-2.44	-1.12
Timor_Leste	TLS	2009	DHS	2016	DHS	-0.28	-0.35	0.28	-0.31	-0.06	-0.67	-0.62	-0.23	-0.05	0.04	-0.96	-0.13
Tanzania	TZA	2010	DHS	2015	DHS	-0.65	0.60	-0.02	0.19	0.03	-0.24	0.04	0.01	1.09	-0.75	0.08	0.15
Uganda	UGA	2011	DHS	2016	DHS	0.42	1.38	0.71	0.81	1.14	0.62	0.45	0.85	1.91	0.97	0.20	1.13
Vietnam	VNM	2010	MICS	2013	MICS	3.60	2.61	0.66	6.47	3.44	2.08				0.18	0.80	3.29
Zimbabwe	ZWE	2009	MICS	2015	DHS	2.22	1.32	-0.04	3.75	1.22	1.23				0.83	0.16	1.65

Figure A7- Average annual percentage points change in child immunization coverage by country and equity group

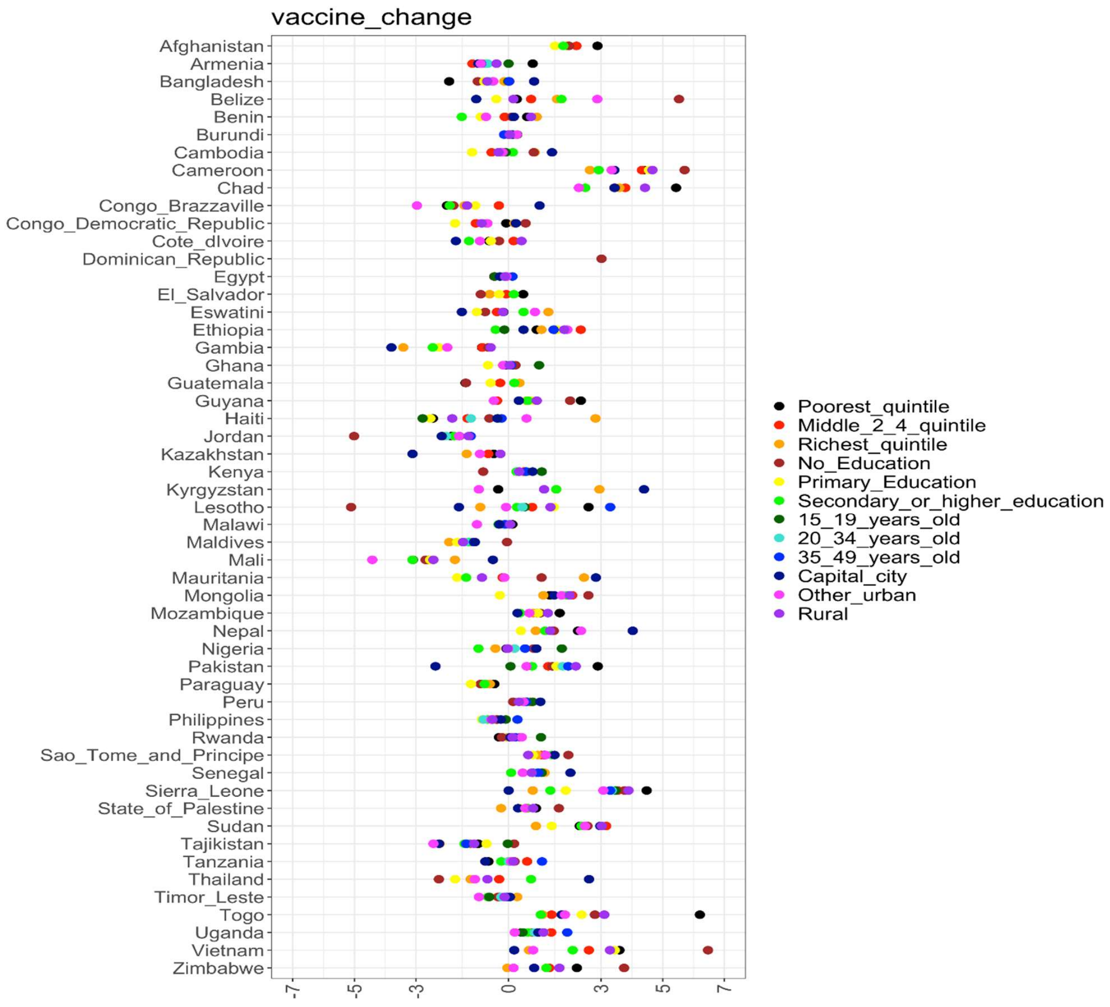

Table A6 – Child illness treatment: Country specific average percentage point change in coverage during the period 2008-2017

country	iso	year_start	source_start	year_end	source_end	Poorest_quintile	Middle_2_4_quintile	Richest_quintile	No_Education	Primary_Education	Secondary_or_higher_education	15_19_years_old	20_34_years_old	35_49_years_old	Capital_city	Other_urban	Rural
Afghanista	AFG	2010	MICS	2015	DHS	-0.33	-0.58	-0.40	-0.68	0.10	-1.12						
Albania	ALB	2008	DHS	2017	DHS	-0.04	-1.38	3.82		-0.90	0.31	1.05	-0.47	-0.62	-2.24	-0.67	-0.51
Armenia	ARM	2010	DHS	2015	DHS	-0.01	5.02	7.18			3.88	-12.08	4.57	4.91		6.76	2.03
Burundi	BDI	2010	DHS	2016	DHS	0.44	0.12	-0.13	0.14	0.08	-0.57	-2.90	0.38	-0.30	-0.32	-0.45	0.18
Benin	BEN	2011	DHS	2014	MICS	-3.95	-5.22	-7.34	-5.67	-4.25	-5.40				-6.97	-5.55	-5.03
Banglades	BGD	2011	DHS	2014	DHS	0.60	1.54	0.03	2.17	0.19	0.65	-1.33	1.38	2.86	-5.83	2.89	1.19
Belize	BLZ	2011	MICS	2015	MICS	0.18	3.18	2.32	10.04	0.85	3.24				1.21	-0.68	3.35
Cote_dIvoi	CIV	2011	DHS	2016	MICS	3.87	-0.44	1.01	0.95	-1.40	1.68				-0.30	0.18	0.96
Cameroon	CMR	2011	DHS	2014	MICS	3.24	-2.12	-2.68	1.81	-0.99	-2.34				-2.69	-2.14	0.34
Congo_De	COD	2010	MICS	2013	DHS	1.21	2.66	2.37	0.81	2.08	3.15				2.75	2.70	2.10
Congo_Bra	COG	2011	DHS	2014	MICS	-4.65	-3.28	-1.56	-1.36	-3.98	-6.22				-2.92	-4.29	-3.94
Cuba	CUB	2010	MICS	2014	MICS					3.17	0.72				1.52	0.41	2.01
Dominican	DOM	2013	DHS	2014	MICS	-3.18	2.86	16.52	-1.83	1.79	3.69				4.47	4.95	-2.65
Egypt	EGY	2008	DHS	2014	DHS	-1.05	-0.09	-1.30	-1.25	-0.34	-0.30	-0.59	-0.39	-0.93	1.25	-1.50	-0.12
Ethiopia	ETH	2011	DHS	2016	DHS	1.82	0.51	-2.80	0.59	0.94	-6.64	2.66	-0.29	2.87	-0.51	0.45	0.66
Ghana	GHA	2008	DHS	2014	DHS	1.91	0.03	-1.09	0.37	1.93	-0.35	-0.36	0.64	0.28	2.64	0.00	0.50
Guinea	GIN	2012	DHS	2016	MICS	-0.18	-0.52	-4.12	-0.54	-4.95	-1.48				-5.16	-5.90	0.46
Gambia	GMB	2010	MICS	2013	DHS	3.45	2.81	4.52	3.81	2.28	3.18				-0.19	4.52	2.13
Guatemala	GTM	2008	RHS	2014	DHS	0.67	1.52	0.62	0.95	1.10	1.35						
Guyana	GUY	2009	DHS	2014	MICS	-0.12	1.13	3.28	0.74	2.57	0.53				7.75	-8.51	0.80
Haiti	HTI	2012	DHS	2016	DHS	-1.38	-1.40	-2.12	0.63	-2.06	-2.10	-5.07	-1.64	-1.00	-0.23	-0.76	-2.17
Jordan	JOR	2012	DHS	2017	DHS	-0.87	0.57	6.66	1.14	-3.37	1.03	-7.77	0.80	1.55	1.18	1.14	-0.51
Kenya	KEN	2008	DHS	2014	DHS	1.53	2.25	2.40	0.98	2.24	1.64	3.59	2.10	1.19	0.83	1.56	2.18
Kyrgyzstar	KGZ	2012	DHS	2014	MICS	-5.83	-2.87	-9.88			-4.28				-12.51	0.07	-3.87
Cambodia	KHM	2010	DHS	2014	DHS	1.90	0.53	-0.80	3.76	-0.14	0.59	-0.05	0.26	2.90	-4.46	1.00	0.83
Lesotho	LSO	2009	DHS	2014	DHS	1.22	-0.69	1.09	8.88	0.17	-0.92	-1.58	-0.21	2.06	1.27	-3.37	0.19
Maldives	MDV	2009	DHS	2016	DHS	1.87	0.94	6.48		1.92	2.71		1.63	1.46	3.41		1.73
Mali	MLI	2009	MICS	2015	MICS	-0.81	-0.96	-2.90	-0.72	-3.23	-3.11				-2.97	-3.20	-0.59
Mongolia	MNG	2010	MICS	2013	MICS	0.38	-0.20	-2.54	2.35	-4.96	-0.73				-1.06	1.24	-0.60
Mozambiq	MOZ	2008	MICS	2015	DHS	-1.26	0.07	0.03	-1.81	-0.18	1.13				1.43	1.16	-0.82
Mauritania	MRT	2011	MICS	2015	MICS	-4.16	-4.82	-1.99	-4.88	-4.54	-5.00				-3.36	-5.71	-4.25
Malawi	MWI	2010	DHS	2015	DHS	1.07	-0.02	-1.57	-0.20	0.22	-1.07	-0.65	0.11	-0.13	0.93	-0.29	-0.02
Nigeria	NGA	2008	DHS	2016	MICS	0.06	-0.85	-2.66	-0.48	-1.45	-2.57	0.06	-0.19	-0.66	-3.08	-0.83	-0.55
Nepal	NPL	2010	MICS	2016	DHS	-0.22	-2.09	-0.69	-1.43	-3.27	-0.42				-3.08	-1.53	-1.84
Pakistan	PAK	2012	DHS	2017	DHS	1.80	1.47	0.27	1.82	0.93	0.11	3.16	0.97	2.85	-0.85	0.61	1.61
Peru	PER	2008	DHS	2016	DHS	-0.90	0.37	-0.41	-1.35	0.04	-0.58	-0.94	-0.11	-0.25	-0.26	-0.34	-0.52
Philippines	PHL	2008	DHS	2017	DHS	1.12	0.72	0.40	7.31	1.77	0.36	-1.64	0.75	1.63	2.58	-0.03	1.19
Paraguay	PRY	2008	RHS	2016	MICS	0.42	-1.49	-0.62	6.89	-0.75	-1.49						
State_of_F	PSE	2010	MICS	2014	MICS	0.73	1.84	1.23	6.43	0.08	2.90				2.36	1.43	1.18
Rwanda	RWA	2010	DHS	2014	DHS	0.54	0.66	-1.31	2.24	-0.09	-0.43	-6.13	0.28	0.75	-0.95	-1.04	0.59
Sudan	SDN	2010	MICS	2014	MICS	-2.22	-1.24	-0.46	-1.53	-1.42	-1.60				-3.53	0.14	-1.24
Senegal	SEN	2010	DHS	2017	DHS	0.61	0.73	-0.02	0.60	0.86	-1.64	0.25	1.04	-0.81	-0.18	0.72	1.01
Sierra_Leo	SLE	2008	DHS	2013	DHS	6.20	3.75	3.74	4.67	2.56	3.39	6.31	3.99	4.66	2.97	2.91	4.60
El_Salvado	SLV	2008	RHS	2014	MICS	3.24	1.74	3.79	-2.03	1.81	1.99						
Sao_Tome	STP	2008	DHS	2014	MICS	-3.67	0.97	-1.52	4.06	-0.64	-0.95				2.27	-2.18	0.09
Eswatini	SWZ	2010	MICS	2014	MICS	3.21	4.77	0.01	5.54	2.88	3.55				8.88	-1.54	5.00
Chad	TCD	2010	MICS	2014	DHS	1.22	1.29	-0.55	0.66	0.95	-2.52				-1.63	-0.48	1.46
Togo	TGO	2010	MICS	2013	DHS	7.65	2.08	2.63	4.25	2.62	4.19				5.23	1.42	4.46
Thailand	THA	2012	MICS	2015	MICS	-5.10	3.40	-1.12	4.43	-1.56	1.54				5.61	-0.18	0.71
Tajikistan	TJK	2012	DHS	2017	DHS	-4.95	1.81	2.54	1.32	2.50	0.67		0.73	2.98	3.90	-0.47	0.77
Timor_Les	TLS	2009	DHS	2016	DHS	0.45	-1.00	1.52	-0.66	0.72	-0.26	-3.18	-0.25	0.77	2.15	-1.22	-0.84
Tanzania	TZA	2010	DHS	2015	DHS	-2.12	-1.50	-1.15	-2.40	-1.20	-2.08	-0.58	-1.33	-2.27	0.54	-2.76	-1.47
Uganda	UGA	2011	DHS	2016	DHS	-0.02	-0.70	-0.08	0.66	-0.79	-0.37	-1.83	-0.30	-0.25	-1.40	0.68	-0.54
Vietnam	VNM	2010	MICS	2013	MICS	0.78	4.30	-1.00	-3.47	3.93	2.61				5.27	1.90	1.78
Zimbabwe	ZWE	2009	MICS	2015	DHS	1.85	3.60	4.77	6.60	1.24	4.14				6.97	3.91	2.72

Figure A8- Average annual percentage points change in child illness treatment coverage by country and equity group

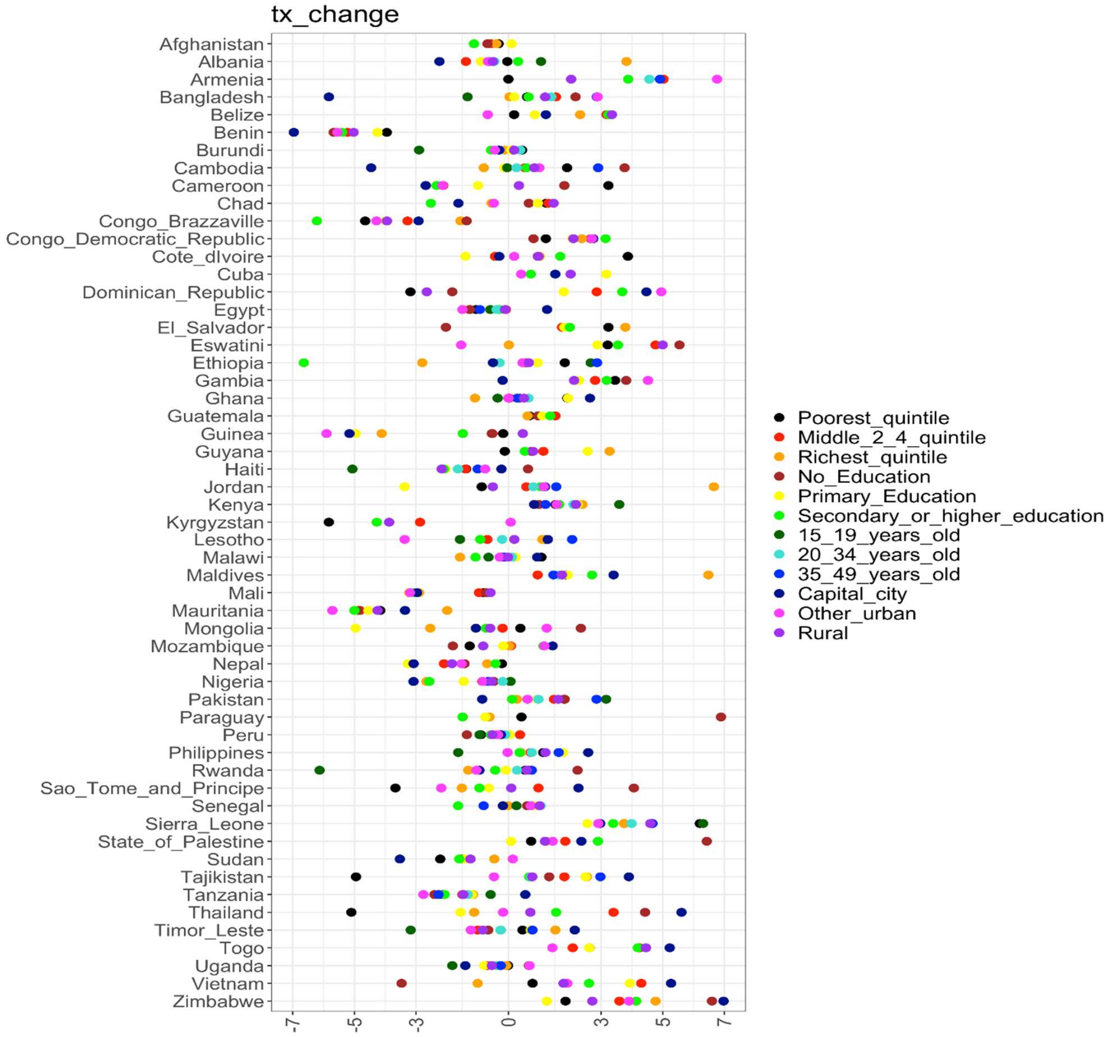

Figure A9: Global coverage gap closed for each of the four components of the continuum of care

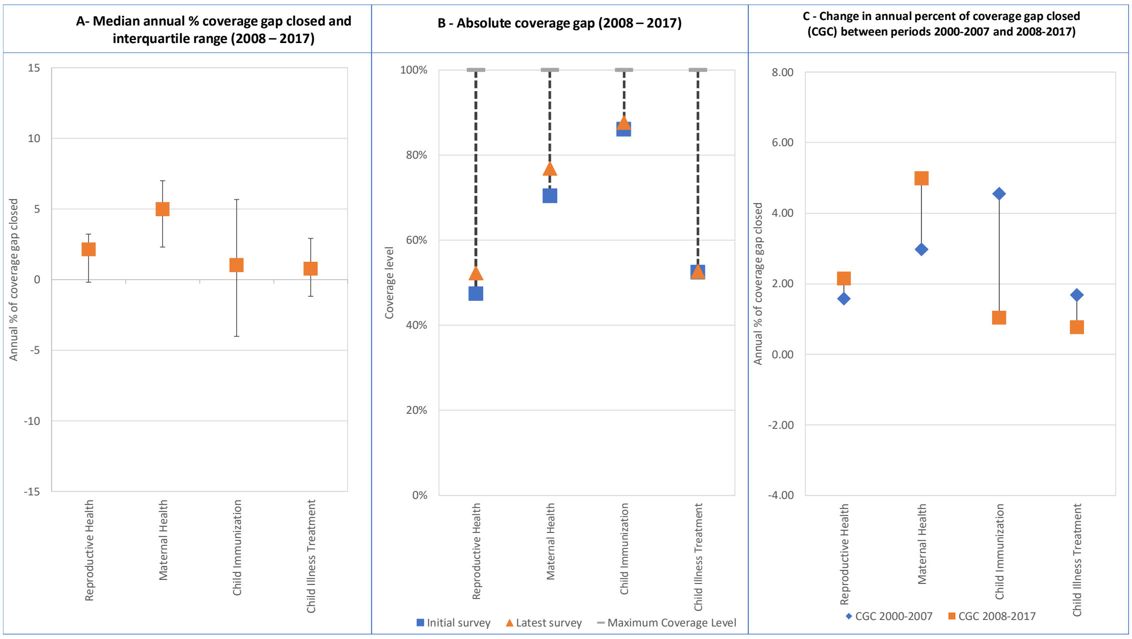

Figure A10: Percent coverage gap closed by country

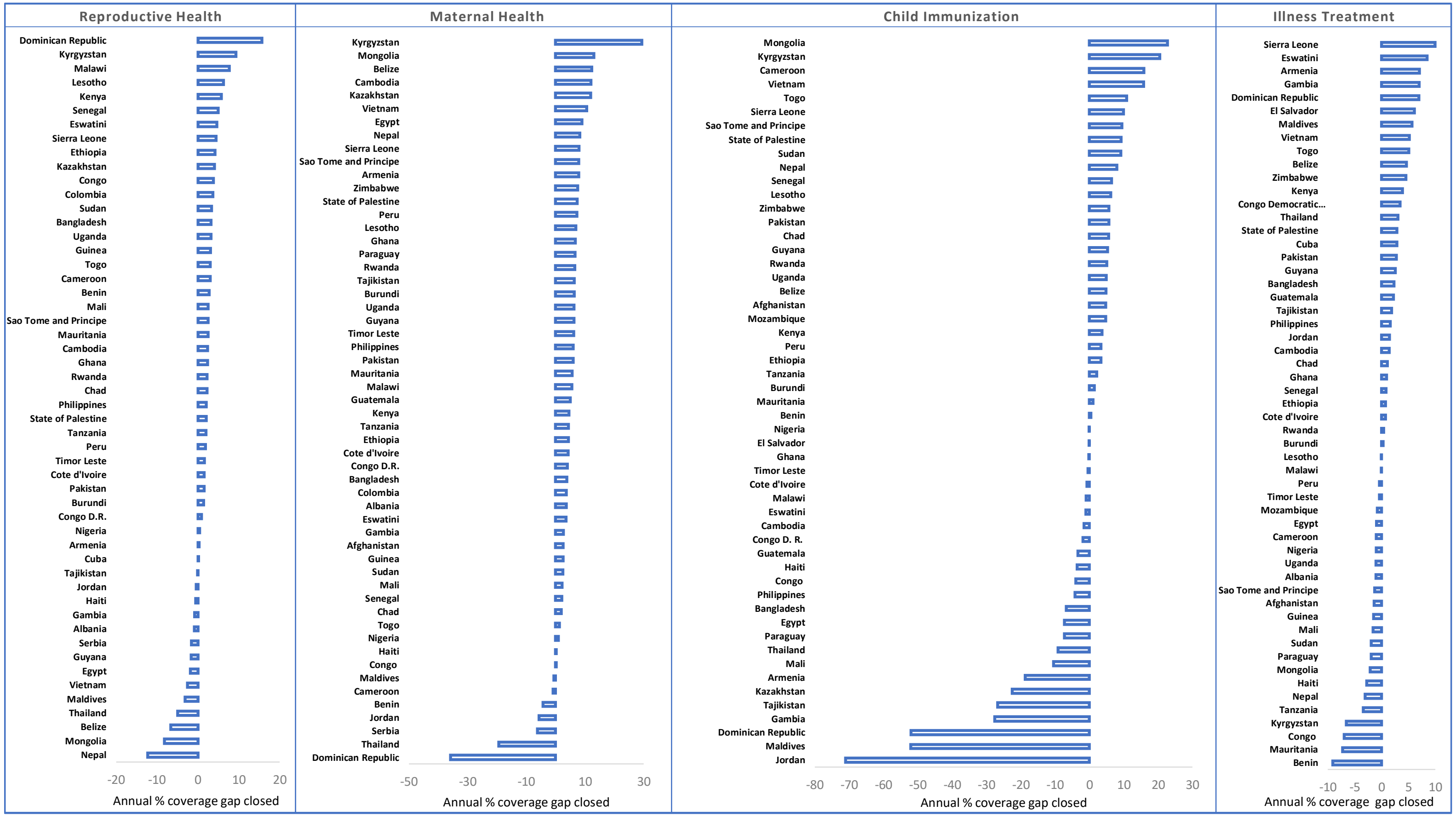

Figure A11: Median and interquartile range of coverage gap closed by specific equity group

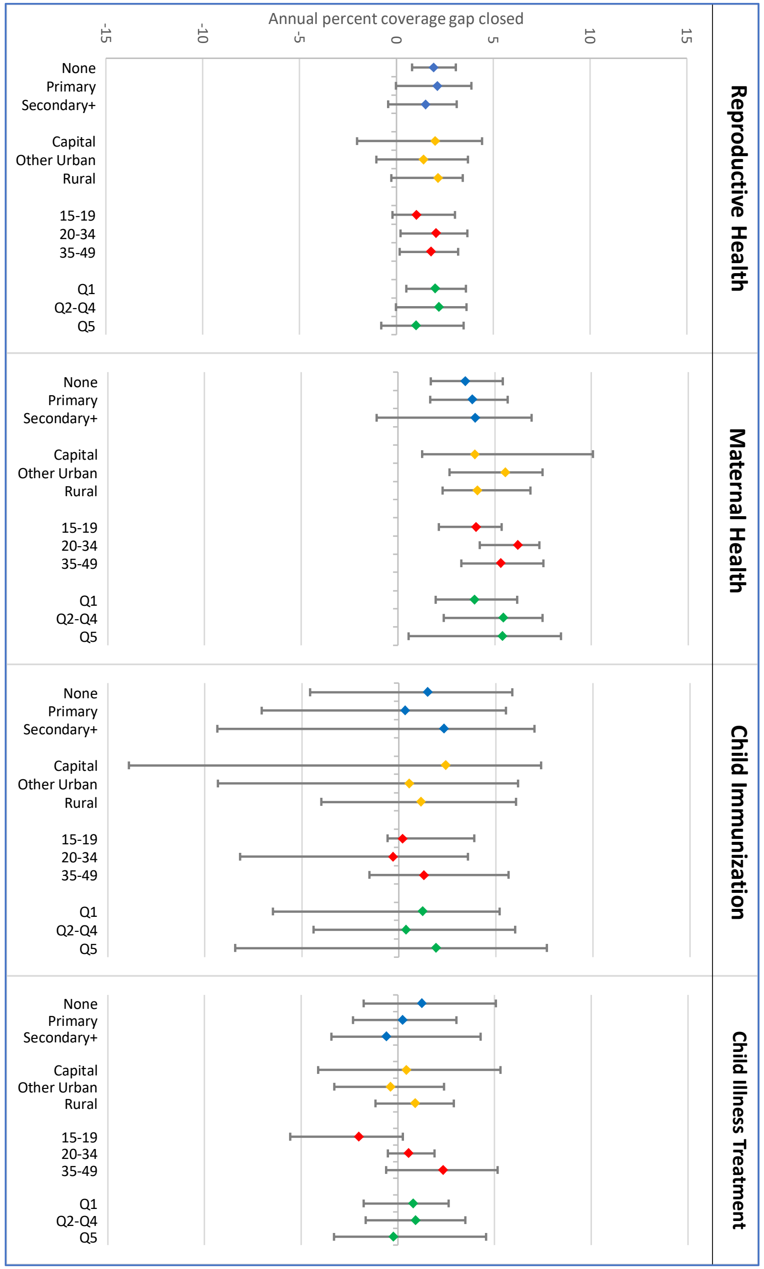

Figure A12: Change in the median and interquartile range of coverage gap closed by specific equity group between the periods 2000-2008 and 2008-2017

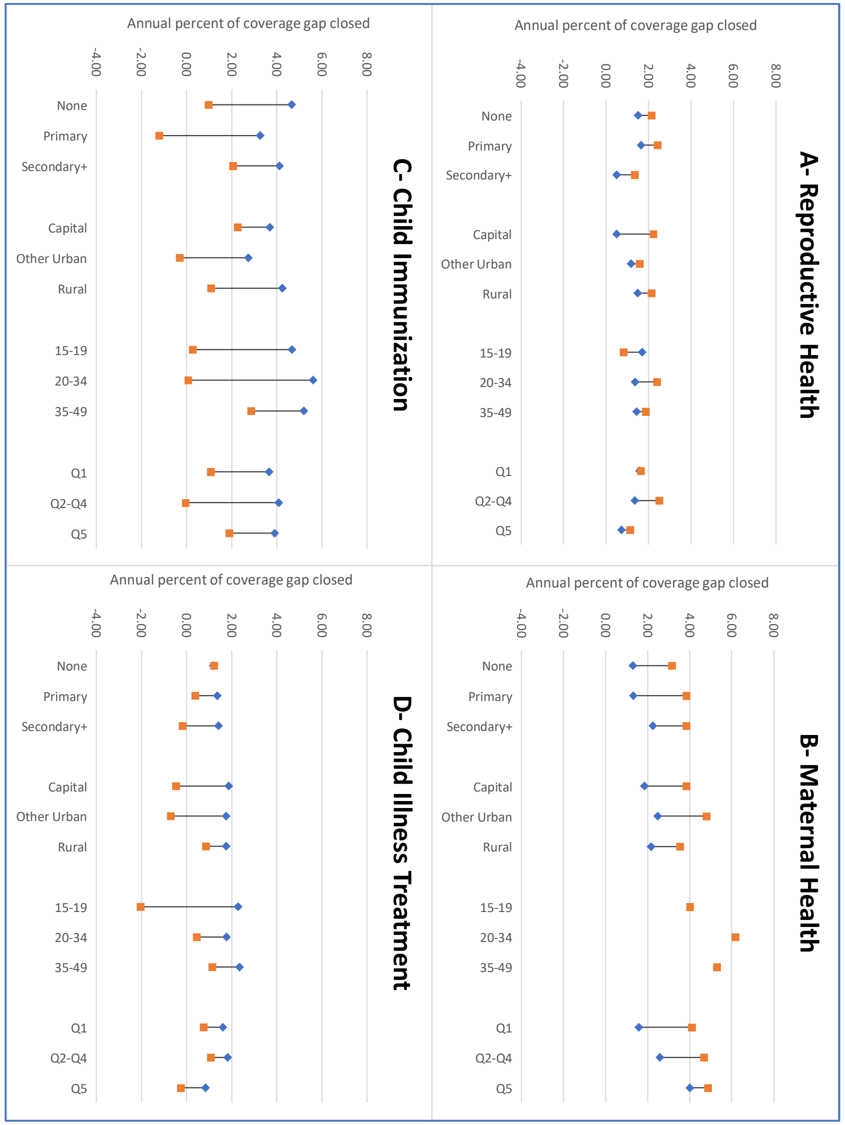

Supplement: Supplementary data [file bmjgh-2019-002230supp001.pdf]
